# Up IGF-I via high-toughness adaptive hydrogels for remodeling growth plate of children

**DOI:** 10.1093/rb/rbaf004

**Published:** 2025-01-23

**Authors:** Zhiqiang Zhang, Haodong Li, Manning Qian, Yiming Zheng, Luhan Bao, Wenguo Cui, Dahui Wang

**Affiliations:** Department of Orthopedics, National Children’s Medical Center & Children’s Hospital of Fudan University, Shanghai 201102, P. R. China; Department of Orthopedics, National Children’s Medical Center & Children’s Hospital of Fudan University, Shanghai 201102, P. R. China; Department of Orthopedics, National Children’s Medical Center & Children’s Hospital of Fudan University, Shanghai 201102, P. R. China; Department of Orthopedics, National Children’s Medical Center & Children’s Hospital of Fudan University, Shanghai 201102, P. R. China; Department of Orthopaedics, Shanghai Key Laboratory for Prevention and Treatment of Bone and Joint Diseases, Shanghai Institute of Traumatology and Orthopaedics, Ruijin Hospital, Shanghai Jiao Tong University School of Medicine, Shanghai 200025, P. R. China; Department of Orthopaedics, Shanghai Key Laboratory for Prevention and Treatment of Bone and Joint Diseases, Shanghai Institute of Traumatology and Orthopaedics, Ruijin Hospital, Shanghai Jiao Tong University School of Medicine, Shanghai 200025, P. R. China; Department of Orthopedics, National Children’s Medical Center & Children’s Hospital of Fudan University, Shanghai 201102, P. R. China

**Keywords:** growth plate, bone bridge, hydrogel, microenvironment, chondrogenic differentiation

## Abstract

The growth plate is crucial for skeletal growth in children, but research on repairing growth plate damage and restoring growth is limited. Here, a high-toughness adaptive dual-crosslinked hydrogel is designed to mimic the growth plate’s structure, supporting regeneration and bone growth. Composed of aldehyde-modified bacterial cellulose (DBNC), methacrylated gelatin (GelMA) and sodium alginate (Alg), the hydrogel is engineered through ionic bonding and Schiff base reactions, creating a macroporous structure. This structure can transform into a denser form by binding with calcium ions. *In vitro*, the loose macroporous structure of the hydrogels can promote chondrogenic differentiation, and when it forms a dense structure by binding with calcium ions, it also can activate relevant chondrogenic signaling pathways under the influence of insulin-like growth factor I (IGF-1), further inhibiting osteogenesis. *In vivo* experiments in a rat model of growth plate injury demonstrated that the hydrogel promoted growth plate cartilage regeneration and minimized bone bridge formation by creating a hypoxic microenvironment that activates IGF-1-related pathways. This environment encourages chondrogenic differentiation while preventing the undesired formation of bone tissue within the growth plate area. Overall, the dual-crosslinked hydrogel not only mimics the growth plate’s structure but also facilitates localized IGF-1 expression, effectively reshaping the growth plate’s function. This approach represents a promising therapeutic strategy for treating growth plate injuries, potentially addressing challenges associated with skeletal growth restoration in pediatric patients.

## Graphical abstract

**Figure:**
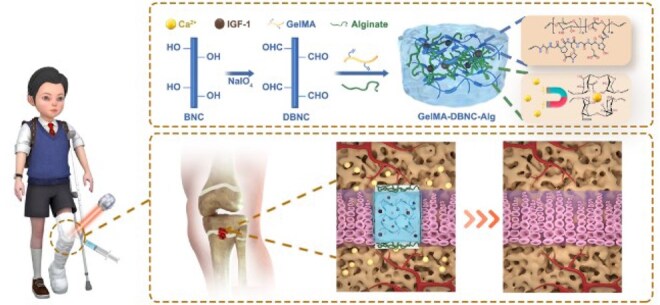

## Introduction

A significant distinction between pediatric and adult bones is the presence of growth plate structures, which facilitate continuous bone growth and development. The growth plate cartilage perpetually generates healthy bone through chondrogenesis [1, 2], increasing bone length. However, the growth plate cartilage is non-renewable and will be replaced by bone tissue rather than cartilage after injury [3–6]. This can impede proper bone growth and even cause deformities. Currently, the treatment for this condition involves surgically removing the bone bridge and employing bone wax or fat implantation to prevent bone bridge reformation. However, the success rate of this procedure is <35% due to inadequate compatibility between the implant materials and the host tissue, often resulting in subsequent complications [1], such as limb length discrepancy, premature epiphyseal closure and limb valgus or varus deformities. Therefore, it is the need of the hour to develop an implant that aligns with the structure and function of the growth plate, preventing the formation of bone bridges, facilitating the regeneration of healthy growth plate tissue, and restoring normal bone growth.

The growth plate’s structure and function depend on the extracellular matrix (ECM), which also influences the fate and status of chondrocytes [7]. The hypoxic microenvironment is critical for the survival of growth plate chondrocytes and the stability of the cartilage matrix. A disruption in the growth plate results in the accumulation of oxygen-rich hematomas at the injury site, leading to an upregulation of osteogenic markers and a downregulation of chondrogenic markers. Consequently, endothelial and osteoblast-like cells proliferate and undergo osteogenic differentiation, ultimately forming a bone bridge [3, 8, 9]. Therefore, the hydrogel material used in this study can rapidly undergo photo-crosslinking to seal growth plate injury sites, simultaneously achieving hemostasis and creating a hypoxic microenvironment. The intricate signaling alterations within the growth plate significantly challenge its reconstruction and treatment. A key growth factor is the growth hormone, a protein secreted by the anterior pituitary gland, which primarily affects the activity of the growth plate by influencing IGF-1 secretion and plays a crucial role in bone and cartilage growth. IGF-1 stimulates chondrocytes to synthesize matrix proteins, such as Col 2 and proteoglycan. It also inhibits chondrocyte degradation and apoptosis following cartilage injury by blocking interleukin-1 or tumor necrosis factor-α [10]. Therefore, the hydrogel used in this study, by incorporating the IGF-1 growth factor, can release IGF-1 in the local hypoxic microenvironment, further promoting the regeneration of growth plate.

**Figure 1.**
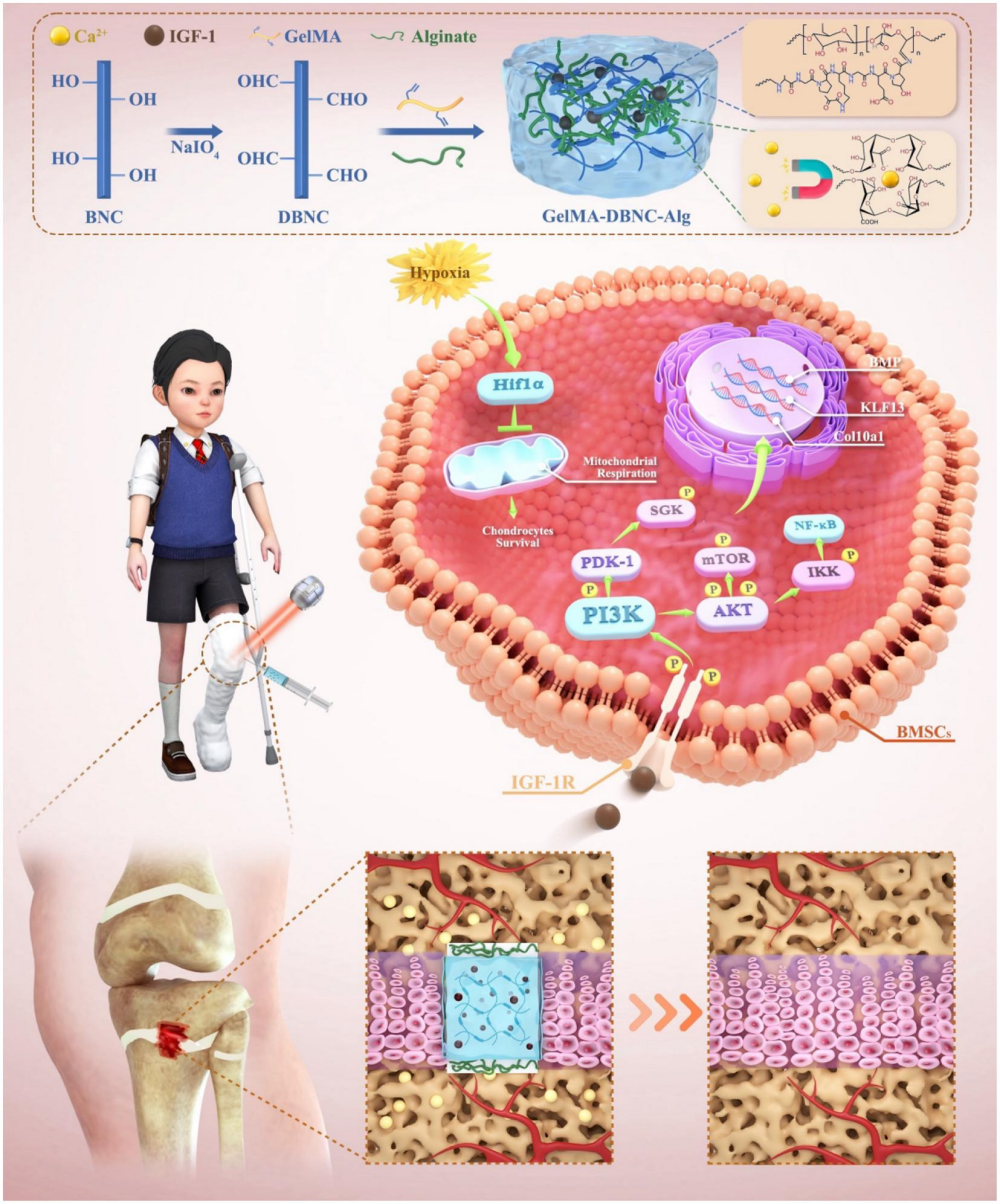
Schematic of injectable hydrogels mimicking the growth plate to enhance bone growth. Injectable high-toughness adaptive dual-crosslinked hydrogels were formulated utilizing GelMA, Alg and DBNC to replicate the layered structure of the growth plate in children. These hydrogels were designed to establish a hypoxic microenvironment by occluding the growth plate damage site and facilitating the overexpression of IGF-1. This strategy aimed to remodel the structure and function of the growth plate, ultimately promoting bone growth.

Limited materials have been explored to restore the structure and function of the growth plate. A study demonstrated inhibiting bone bridge formation following sheep growth plate injury using a gelatin sponge scaffold combined with transforming growth factor (TGF)-β1 and autologous bone marrow mesenchymal stem cells (BMSCs) [11]. Guan et al. [12] synthesized a chondroitin sulfate-methacrylic anhydride (MA) gelatin hydrogel with exosomes. They implanted it on the damaged area of the growth plate, suppressing the inflammatory response and promoting cartilage regeneration. However, most previous implant studies focused solely on filling the growth plate defect without considering its physiological structure and function. Since the growth plate is responsible for longitudinal growth and bears weight between the epiphysis and metaphysis, an ideal bionic scaffold should possess excellent biocompatibility, biodegradability, appropriate porosity and suitable mechanical properties [13]. To date, most hydrogels are mechanically weak and brittle, the recently reported double-network hydrogel achieves high toughness by effectively dissipating mechanical energy through the addition of sacrificial bonds, which provides hope for the application of hydrogels [14]. Inspired by that, hydrogels with extremely high mechanical strength and toughness can be constructed by forming an interpenetrating network of two polymers with different properties. The fracture “sacrifice bond” is used to effectively dissipate energy, thereby enhancing the mechanical properties of hydrogels. Therefore, adding cellulose to the hydrogel was reported to significantly enhance the mechanical properties of the scaffold [15, 16]. This study creatively utilized the Schiff base bonding of GelMA and DBNC and the electrostatic attraction of ionic bonding between Alg to construct high-toughness adaptive dual-crosslinked hydrogels that mimic the hierarchical structure of pediatric growth plates. These hydrogels mediated IGF-1 overexpression in the microenvironment and remodeled the growth plate structure and function (Figure 1). Biocompatibility tests confirmed the suitability of the hydrogel, which exhibited chondrogenic functionality before calcium ion recruitment and osteogenic functionality after calcium ion recruitment. Furthermore, a rat growth plate injury model was evaluated by magnetic resonance imaging (MRI). RNA-Seq analysis identified differentially expressed genes (DEGs), shedding light on the characteristics of the symbiotic ecological niche. Histomorphological analysis elucidated the regulatory effects of hydrogels on tissue regeneration processes, cellular functions and signal transduction. This study will build a foundation for future research to utilize specific hydrogel materials to regenerate growth plate cartilage effectively.

## Materials and methods

### Synthesis of GelMA

Twenty grams of gelatin was dispersed in 200 ml of carbonate buffer solution (pH 9.0) prepared by dissolving Na_2_CO_3_ (0.3427 g) and NaHCO_3_ (3.0915 g) in 200 ml of deionized water. The mixture was placed in a 50°C oil bath and stirred until complete dissolution, resulting in a 10% gel solution. Two milliliters of MA was drawn into a syringe and slowly added to the gel solution at a rate of 0.2 ml/min using a micro syringe pump while protecting the solution from light. The reaction was carried out in an oil bath for 3 hours. Subsequently, 100 ml of PBS was added to terminate the reaction. After that, the unreacted MA was removed by centrifugation at 7000 rpm for 15 minutes; the resulting GelMA was dispensed into dialysis bags (MWCO 3500) and dialyzed at 38°C for 2 days. Next, the samples were freeze-dried to obtain the final product.

### The preparation of BNC and synthesis of DBNC

The preparation process was reported previously [17]. The fermentation medium, containing 100 g/l D-fructose, 5 g/l peptones and 3 g/l yeast extract, was adjusted to pH 5.0–5.2 with citric acid, sterilized at 115°C for 30 minutes, and cooled. Komagataeibacter xylinus (K.xylinus, China General Microbiological Culture Collection Center) was inoculated and incubated at 30°C, 160 rpm overnight. Then, 1 ml of broth was added to each well of a 24-well plate and cultured statically at 30°C for 14 days to obtain BNC at the gas–liquid interface. The BNC was boiled in 1% NaOH at 80°C for 4 hours, washed to neutral pH, and sterilized at 121°C for 30 minutes (5 cycles). It was stored in ultrapure water for use.

The synthesis was performed as previously reported [18]. Briefly, 1 g of BNC dry film was weighed, cut into pieces and homogenized in 200 ml of deionized water. The resulting mixture was transferred to a 500-ml round-bottomed flask wrapped with tin foil to protect it from light. Next, NaIO_4_ oxidant was added in a ratio of 5:10 (*w*: NaIO_4_/*w*: BNC), and the reaction was carried out at 40°C and pH 6.0 for 14 hours. After the reaction, the precipitate was filtered and collected. 0.1 mol/l ethylene glycol solution was added to collect the precipitate, and the reaction was continued for more than 30 minutes to remove the unreacted NaIO_4_. The resulting precipitate was filtered and washed five times with deionized water. The filter residue was collected and freeze-dried to yield DBNC.

### Assembling the hydrogels

A specific amount of GelMA was taken and dissolved in ultrapure water to prepare a hydrogel solution with a 5–10% (w/v%) concentration, Irgacure 2959 was added with 0.5% concentration. Subsequently, a specific amount of DBNC was dissolved in the hydrogel solution to create a mixed hydrogel with a concentration ranging from 0.5% to 1%. Next, a specific amount of Alg was dissolved in the mixed hydrogel solution to achieve a concentration of 1.2% following the procedure described in the literature [19]. The various materials were mixed using an ultrasonic cell breaker and cross-linked in the presence of UV light.

### Determining the material concentration

The prepared hydrogels (GelMA-DBNC-Alg) were divided into nine groups and tested for mechanical properties. The elongation at break curves was plotted, and Young’s modulus was calculated based on the results. The differences in mechanical properties between the groups were analyzed, and suitable concentrations of various materials were selected. The concentration of IGF-1 was 100 ng/ml [20].

### Characterization of different groups of hydrogels

The hydrogels’ microstructure and cells’ morphology were observed using scanning electron microscopy (SEM). The chemical structures of the different groups of hydrogels were analyzed after lyophilization using attenuated total reflection-Fourier transform infrared spectroscopy. The swelling rates of the hydrogels were determined by immersing the lyophilized materials in ultrapure water at 37°C with agitation at 80 rpm. The mass of the hydrogels was measured at various time points, such as 2, 8, 24, 48, 72 and 96 hours. The release of IGF-1 from the GDAI at different time points was quantified using enzyme-linked immunosorbent assays to determine the encapsulation rate and release properties of IGF-1. Three parallels were set.

The formula was calculated as follows: cumulative release %=Mt/M * 100%

where Mt represents the amount of IGF-1 released at time t, and M represents the total amount of IGF-1 in the hydrogel.

### Biocompatibility and effects on cell morphology

Different materials, including GelMA+DBNC (GD), GelMA+DBNC+Alg (GDA) and GelMA+DBNC+Alg+IGF-1 (GDAI), were placed in 24-well plates with and without calcium, and three parallel controls for each material. Next, the cross-linking was done, followed by UV sterilization for over 2 hours. Third-generation rat BMSCs, at a density of 1.2 × 10^5^ cells/ml, were seeded on the samples and incubated in α-MEM supplemented with 10% fetal bovine serum and 1% penicillin/streptomycin at 37°C with 5% CO_2_ and 21% O_2_ for 1, 3 and 5 days. The BMSCs planted in the plate without any intervention were labeled as blank control. Post-incubation, the samples were washed thrice with PBS and co-incubated with a mixture of 40 μl CCK-8 and 360 μl α-MEM for 1 hour. Subsequently, 100 μl of the cell suspension was transferred to a 96-well plate, and the absorbance was recorded at 450 nm to determine the cytotoxicity of the material. The results were presented as the mean ± standard deviation. The cells were fixed after 5 days of co-culture to examine the morphology of the individual cells after co-culture; SEM was employed to observe the cells. The biocompatibility of the materials was assessed by live-dead cell staining after 24 and 48 hours of co-culture using Transwell plates.

### Transcriptome analysis

Different groups were co-cultured with BMSCs for 7 days, followed by transcriptome analysis. Samples from the control group, GDAI group and GDAI+Ca^2+^ group were pulverized under liquid nitrogen. The RNAmini kit (Qiagen, Germany) was used to extract the total RNA; the RNA quality was examined by gel electrophoresis and Qubit (Thermo, Waltham, MA, USA). Strand-specific libraries were constructed using the TruSeq RNA sample preparation kit (Illumina, San Diego, CA, USA), and sequencing was carried out using the Illumina Novaseq 6000 instrument, according to the manufacturer’s protocol. Servicebio Technology (Wuhan, China) was used for transcriptome sequencing. The expression levels of transcripts were quantified using Perl’s FPKM. Differentially expressed transcripts were determined using the MA-plot-based random sampling method implemented in the DEGseq package. DEGs, Gene Ontology (GO) and Kyoto Encyclopedia of Genes and Genomes (KEGG) enrichment analyses were performed using R software. Statistical significance was defined using *P*-values <0.05 and fold change ≥2 thresholds.

The protein–protein interaction (PPI) network analysis of DEGs was based on the STRING database, where known and predicted protein–protein interactions are available. Networks were created based on known interactions of selected reference species.

### Quantitative real-time polymerase chain reaction analysis and Western blot analysis

To verify whether different groups of hydrogels can promote cartilage regeneration. The results of polymerase chain reaction (PCR) were normalized to GAPDH and analyzed using the 2^−△△Ct^ method. The primer sequences are listed in Supplementary Table S1.

Western blot (WB) was used to validate the preliminary results of the transcriptome analysis and identify the essential proteins of the relevant pathways. PCR and WB experiments were performed as previously reported [21].

### Animal model of growth plate injury in rats

All surgical procedures and perioperative management were performed in accordance with the protocols approved by the Animal Research Committee of the Children’s Hospital of Fudan University (2023-EKYY-121JZS). Twenty, ~ 5-week-old male rats were prepared and divided equally into blank control, GD, GDA and GDAI groups.

According to the literature, a rat model of proximal tibial growth plate injury was prepared [22]. Briefly, the rats were anesthetized by isoflurane, and the animals were moved to the surgical site to lie flat on a warming pad and an absorbent pad. An electric razor shaved off the entire hind leg hair from the medial ankle to the pelvis. The surgical site was cleaned by wiping the entire leg, abdomen and genitalia with an alcohol swab, followed by povidone-iodine-soaked gauze. Subsequently, a cavity towel was laid, and a 2-cm incision was made distally, beginning from the inferior border of the patella. The tibial fascia and soft tissues were gently dissected or scraped with a scalpel, and a 1.6-mm Kirschner needle was inserted perpendicular to the tibial tuberosity and slowly drilled to a depth of about 1 cm; Care was taken not to insert the needle too deep. A gauze was applied to stop bleeding, followed by injection and cross-linking of the hydrogel. The hydrogels were cross-linked through a UV-induced photopolymerization process. Finally, the wound was sutured (Supplementary Figure S1).

### Animal MRI examination and immunohistochemical staining

In vivo, MRI at 8 weeks after surgery assessed the growth plate damage and regeneration with the following settings.

T_1_-weighted: SE (TE/TR = 10/300, FOV = 40 × 20 mm, matrix = 256 × 128, NEX = 10, Res. 156 μm, Acq. time 6:42 minutes); T_2_-weighted SE (TE/TR = 73.8/3100, FOV = 40 × 20 mm, matrix = 256 × 128, NEX = 20, ETL = 16, Res. 156 μm, Acq. time 10:00 minutes).

The rats were euthanized after 9 weeks, and proximal tibial specimens were collected for immunohistochemical staining. The collected specimens were fixed and decalcified with 10% formic acid at room temperature for 2 weeks. Subsequently, the specimens underwent ethanol gradient dehydration and paraffin block embedding. The embedded specimens were then sectioned into 5-μm-thick slices precisely through the center of the defect area and stained with hematoxylin and eosin (H&E) and toluidine blue (TB). The extent of cartilage damage and the regeneration of the growth plate could be determined via this process.

### Transcriptome analysis

Rat growth plate tissues from the control and GDAI groups were taken and subjected to RNA-seq assay at 9 weeks after surgeries. The specific steps are listed above like cells transcriptome analysis. PCR assays were performed to validate the results of RNA-seq.

### Statistical analysis

Graphpad prism (v9.0 CA) was used for statistical analysis of the experimental results. All data were expressed as mean ± standard deviation. Multiple experimental groups were analyzed using one-way analysis of variance. R software was used for statistical analysis of the RNA-seq results. The statistical tests performed in this study are shown in the figures: *0.01<*P* < 0.05; **0.001<*P* < 0.01; ***0.0001<*P* < 0.001; *****P < 0.0001.

## Results

### Characterization of high-toughness hydrogels

**Figure 2.**
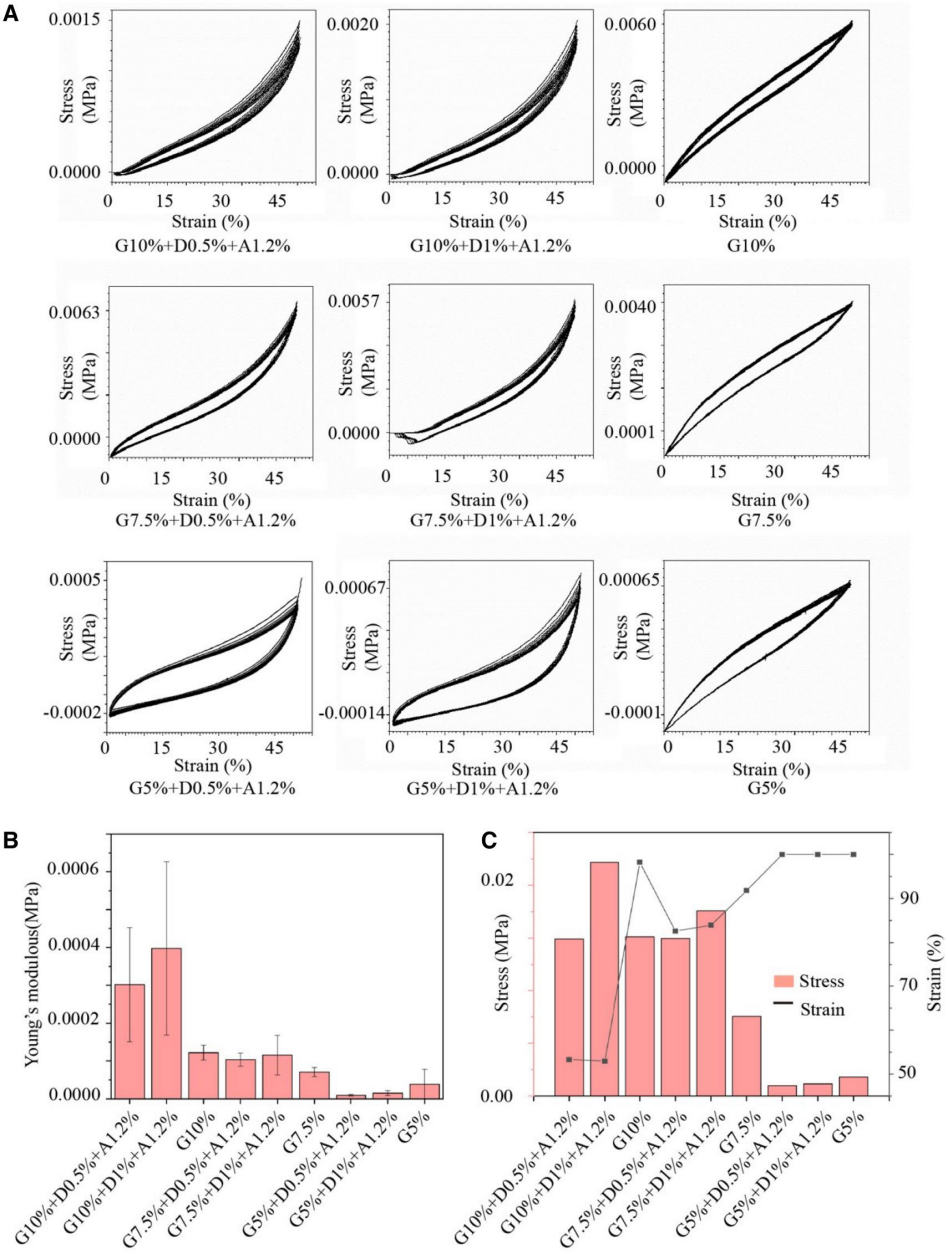
Mechanical behavior of hydrogels with different material concentrations. The repeated compression tests (**A**), the Young’s modulus (**B**) and the strain and stress (**C**).

Recent studies have highlighted the promising properties of Alg hydrogels for promoting chondrocyte proliferation, adhesion and migration. Hydrogels exhibit enhanced biological functions when Alg and calcium ions are maintained at 1.2% and 102 mM [23–27]. To further determine the concentration of GelMA and DBNC, repeated compression tests were conducted on hydrogels with different material concentrations for 10 cycles to assess their mechanical behavior, and stress–strain diagrams were generated based on the results (Figure 2A). Data analysis revealed that with a gradual increase in the concentration of GelMA, while keeping the concentration of other materials constant, the hydrogel demonstrated increased resistance during the deformation process at 50%, indicating improved mechanical strength. Conversely, no significant changes were found regarding stress at a constant concentration of GelMA with a gradual increase in the concentration of DBNC. Next, Young's modulus and maximum stress–strain results of the hydrogels at different concentrations were calculated (Figure 2B and C). The GelMA concentration of 7.5% retained mechanical strength while allowing for optimal elastic deformation of 85%. An increase in the DBNC concentration enhanced the overall Young’s modulus and mechanical properties of the hydrogel. Based on these experimental findings, the hydrogel material was recommended to be composed of 7.5% GelMA, 1% DBNC, and 1.2% Alg.

**Figure 3.**
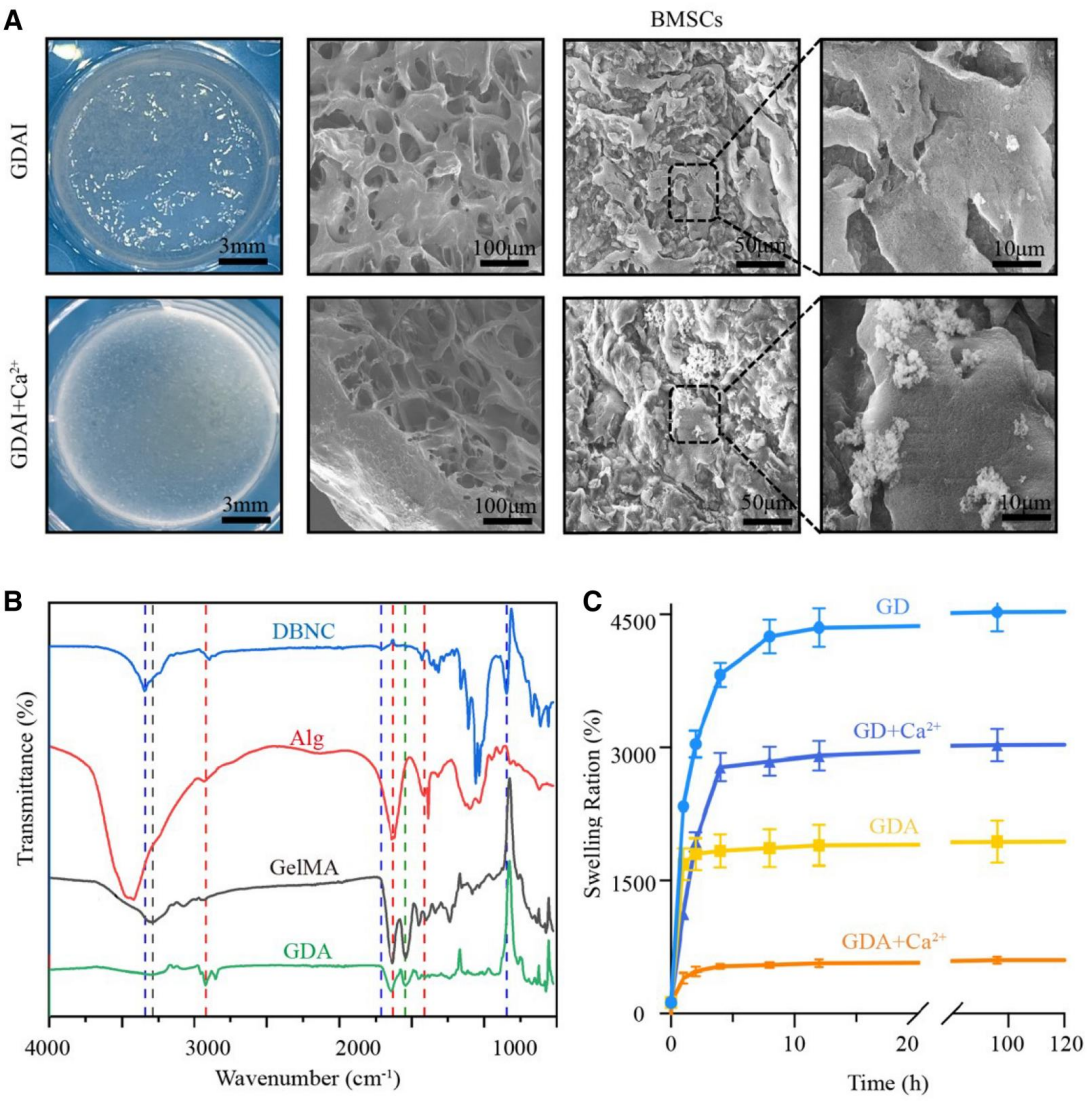
Micromorphology and characteristics of hydrogels. SEM images of hydrogels with or without BMSCs (**A**), FTIR (**B**) and the swelling ration of hydrogels (**C**).

In this study, the formation of a Schiff base bond (DBNC-CH=N-GelMA) between the amino group (GelMA hydrogel) and the aldehyde group (DBNC) resulted in the formation of the first layer of the structure (Supplementary Figure S2). Subsequently, adding Alg material facilitated the recruitment of free calcium ions from the metaphysis, leading to sustained cross-linking [28]. The internal structure of the hydrogel was further examined via SEM. Figure 3A provides insights into the internal fibrous structure of the hydrogels when DBNC was added. In the presence of calcium ions, the hydrogel surface displayed evident cross-linking, characterized by smoother and denser internal connectivity, compared with the macroporous internal structure (Supplementary Figure S3). The microscopic morphology exhibited denser changes after the addition of Alg (Supplementary Figure S4). These findings confirmed that this hydrogel could form a dense structure on the surface upon contact with metaphysis by recruiting calcium while maintaining a loose and macroporous internal structure. Next, SEM was done to investigate the growth morphology of BMSCs on GDAI hydrogels. BMSCs exhibited a flattened morphology on the hydrogels. In the presence of calcium ions, it was observed that BMSCs secreted more calcium and phosphorus nodules (Figure 3A).

The FTIR results (Figure 3B) showed that the spectrum of DBNC exhibited characteristic peaks corresponding to different vibrational modes. The peaks observed at approximately 3342 cm^−1^ were attributed to hydroxyl (O–H) stretching vibrations, while those at 292 cm^−1^ corresponded to C–H stretching vibrations. The peaks around 1020 cm^−1^ represented C–O stretching vibrations of hydroxyl groups, and the peaks at 1730 cm^−1^ indicated carbonyl (C=O) stretching vibrations. Additionally, the peak at 880 cm^−1^ suggested hemiacetal stretching. These observations collectively confirmed the successful oxidation of the hydroxyl group at the C2 and C3 positions of BNC to an aldehyde group via the action of sodium periodate [18]. Notable changes were observed in the spectrum of GDA, which was generated from the reaction between DBNC and GelMA via Schiff base formation. The characteristic peak of –CHO in DBNC disappeared at 1720 cm^−1^, and the absorption peak corresponding to the amide A band in GelMA disappeared in the 3400–3500 cm^−1^ range. Furthermore, a characteristic peak near 1549 cm^−1^ indicated the presence of the –C=N imine bond in the resultant product. These findings suggested that the –CHO group in DBNC reacted with the –NH group in GelMA, forming the Schiff base bond [29].

Figure 3C shows that the addition of Alg to the GD hydrogel resulted in a significant reduction in its swelling performance. This change was attributed to alterations in the internal macropore structure and enhanced internal connectivity of the material. Incorporating calcium ions further tightened the hydrogel, leading to a much lower swelling rate at different time points than in the absence of calcium ions. In the absence of Alg, adding calcium ions still contributed to a reduced swelling rate. This effect was attributed to the cross-linking of the internal negative charge of DBNC with the positive charge of calcium ions [30, 31]. Supplementary Figure S5 presents the release curve of IGF-1, revealing an initial burst release effect within the first 12 hours, followed by a relatively gradual release. Additionally, it was evident that the release rate of IGF-1 was lower in the material group containing calcium ions compared to the group without calcium ions in the first 3 days. This finding indicated that the hydrogel will gradually become a dense structure and then enable a slower release of IGF-1.

### Biocompatibility and chondrogenic/osteogenesis assay *in vitro*

**Figure 4.**
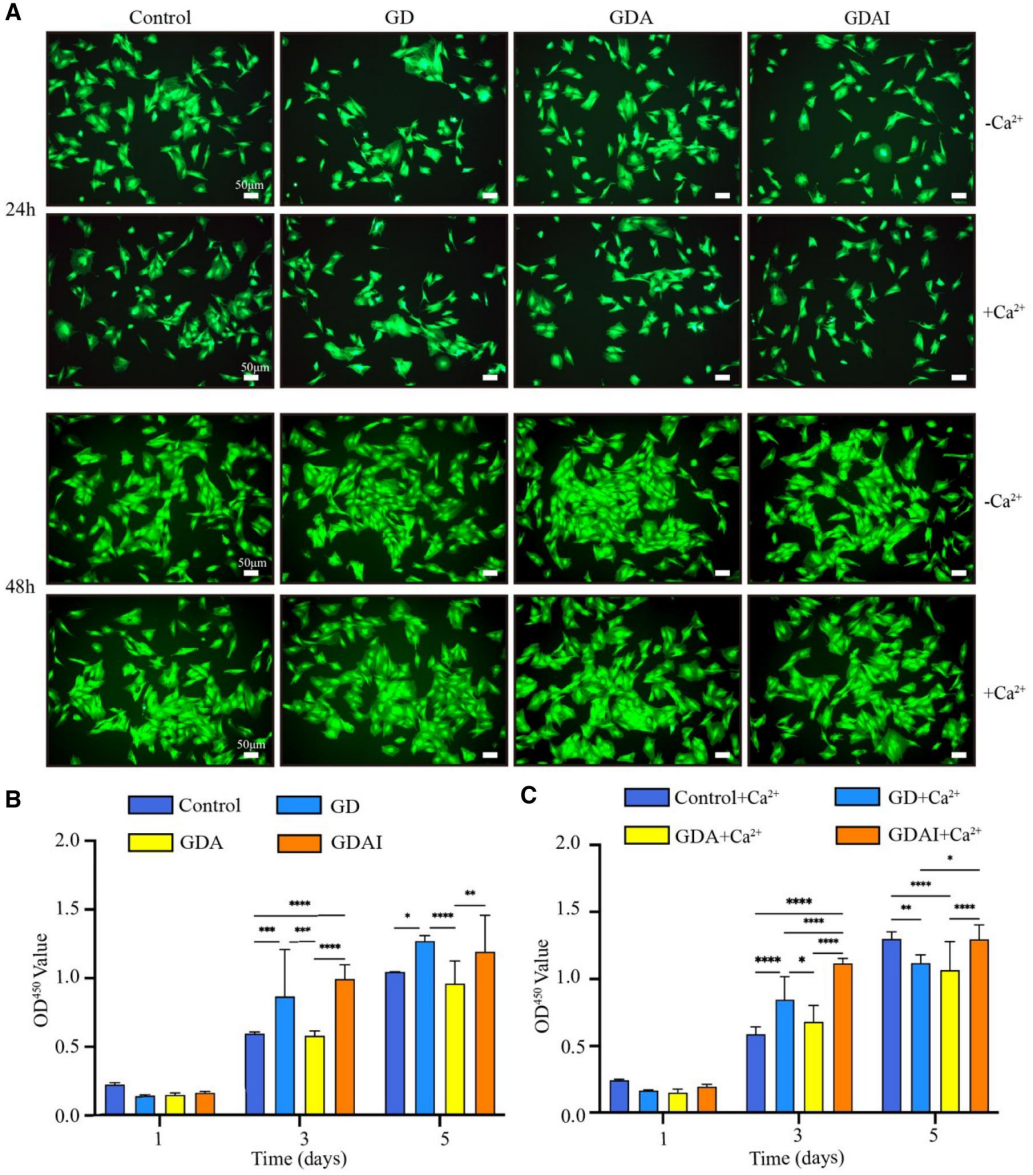
Biocompatibility of hydrogels with or without Ca^2+^. Live/dead staining of BMSCs (**A**), CCK-8 results of BMSCs (**B**), CCK-8 results of BMSCs+Ca^2+^ (**C**) The asterisk (*) represents a significant difference (*P < 0.05, **P < 0.01, ***P < 0.001, ****P < 0.0001).

The results of live-dead staining of BMSCs after 24 and 48 hours of co-culture with the hydrogel demonstrated robust cell growth without any apparent cell death, indicating excellent biocompatibility of the material (Figure 4A). Furthermore, the CCK-8 assay revealed the proliferation and survival of BMSCs in the presence and absence of calcium ions (Figure 4B and C). There was no significant difference in control and three scaffold groups co-cultured for 1 day, and the proliferation of BMSC was significant when co-cultured with scaffolds for 3 and 5 days. In a word, hydrogels all appear excellent biocompatibility *in vitro*.

**Figure 5.**
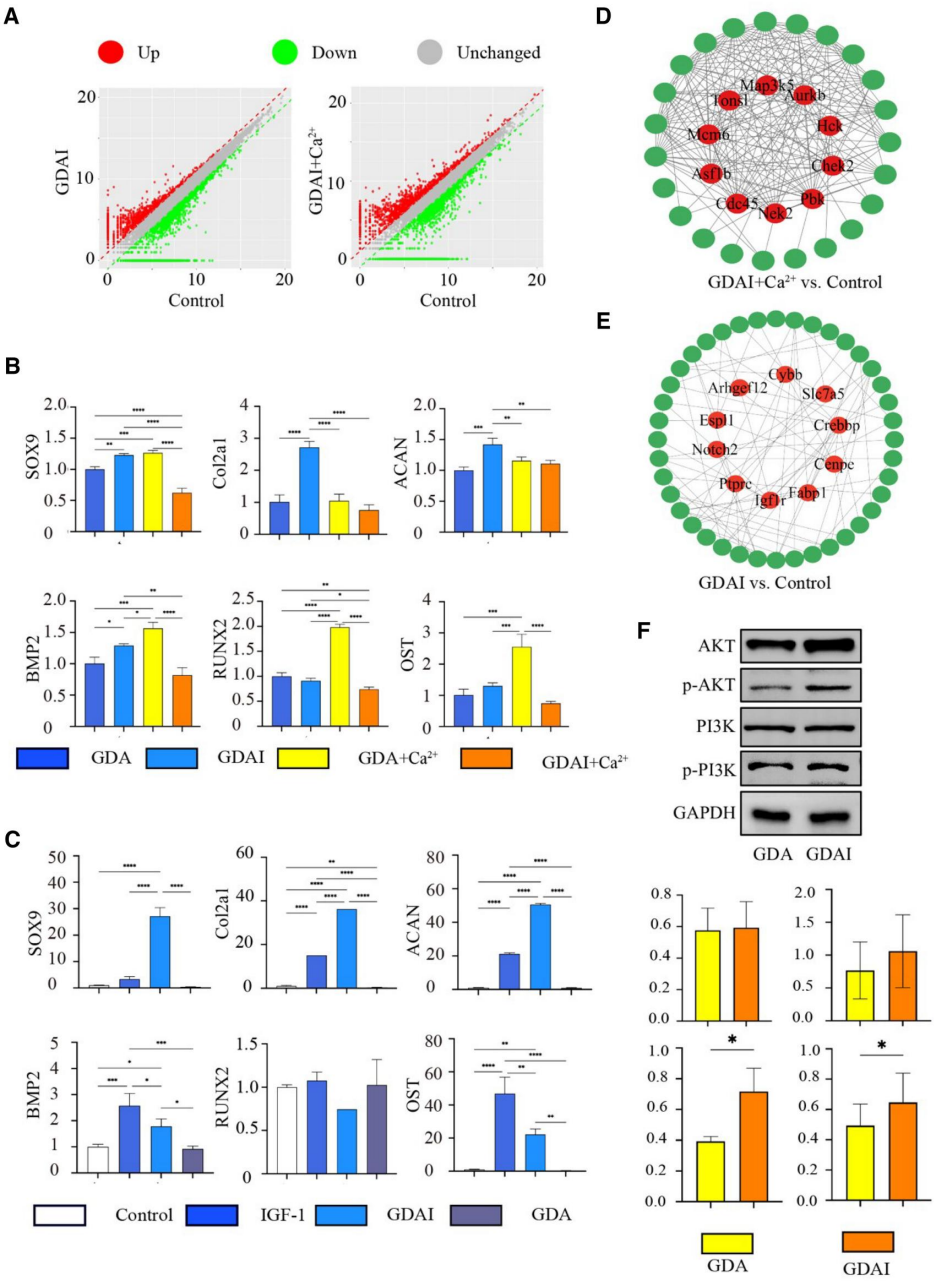
Transcriptome analysis of hydrogels function on BMSCs. The DEGs in hydrogels (**A**), validation of chondrogenic and osteogenic differentiation of BMSCs by RT-qPCR (**B** and **C**), the string network of DEGs between groups (**D** and **E**), validation of KEGG pathway (upregulated DEGs) by Western blot (**F**), DEGs were presented with FoldChange > 2 or FoldChange < 0.5. The asterisk (*) represents a significant difference (*P < 0.05, **P < 0.01, ***P < 0.001, ****P < 0.0001).

BMSCs were co-cultured with the control group, GDAI group and GDAI+Ca^2+^ group for 7 days, followed by RNA-seq to identify DEGs to confirm the differentiation-inducing effect of the hydrogels under specific conditions. (Figure 5A) In order to explore the effect of IGF-1 and calcium ion on the material. We have four groups of materials for osteogenesis and chondrogenic genetic testing (Figure 5B). The expressions of SOX9, ACAN and Col2a1 were upregulated in the GDAI compared with the GDA group, but no significant change on the RUNX2 and OST, indicating that the IGF-1 enhanced the chondrogenic differentiation ability of BMSCs. When comparing the GDA+Ca^2+^ group with the GDA group, BMP2, RUNX2 and OST were significantly increased, indicating that the osteogenic ability of the material was enhanced under the effect of calcium ions. However, in the presence of IGF-1 (GDAI vs. GDAI+Ca^2+^), the osteogenic ability promoted by calcium ions was weakened. In the presence of calcium ions (GDA+Ca^2+^ vs. GDAI+Ca^2+^), IGF-1 reduced the osteogenic potential of the scaffold (BMP2, RUNX2 and OST were all significantly decreased). The above results confirmed that the addition of the IGF-1 growth factor to the hydrogel could promote the chondrogenic differentiation of BMSCs and reduce their osteogenic differentiation ability. We further compared the osteogenic and chondrogenic differentiation of BMSCs in the blank group, IGF-1 group, GDA group and GDAI group. IGF-1 could significantly increase the expression of osteogenic or chondrogenic genes, but when IGF-1 was combined with GDA material, the chondrogenic differentiation ability was significantly improved and the osteogenic differentiation ability was decreased compared with other groups (Figure 5C).

**Figure 6.**
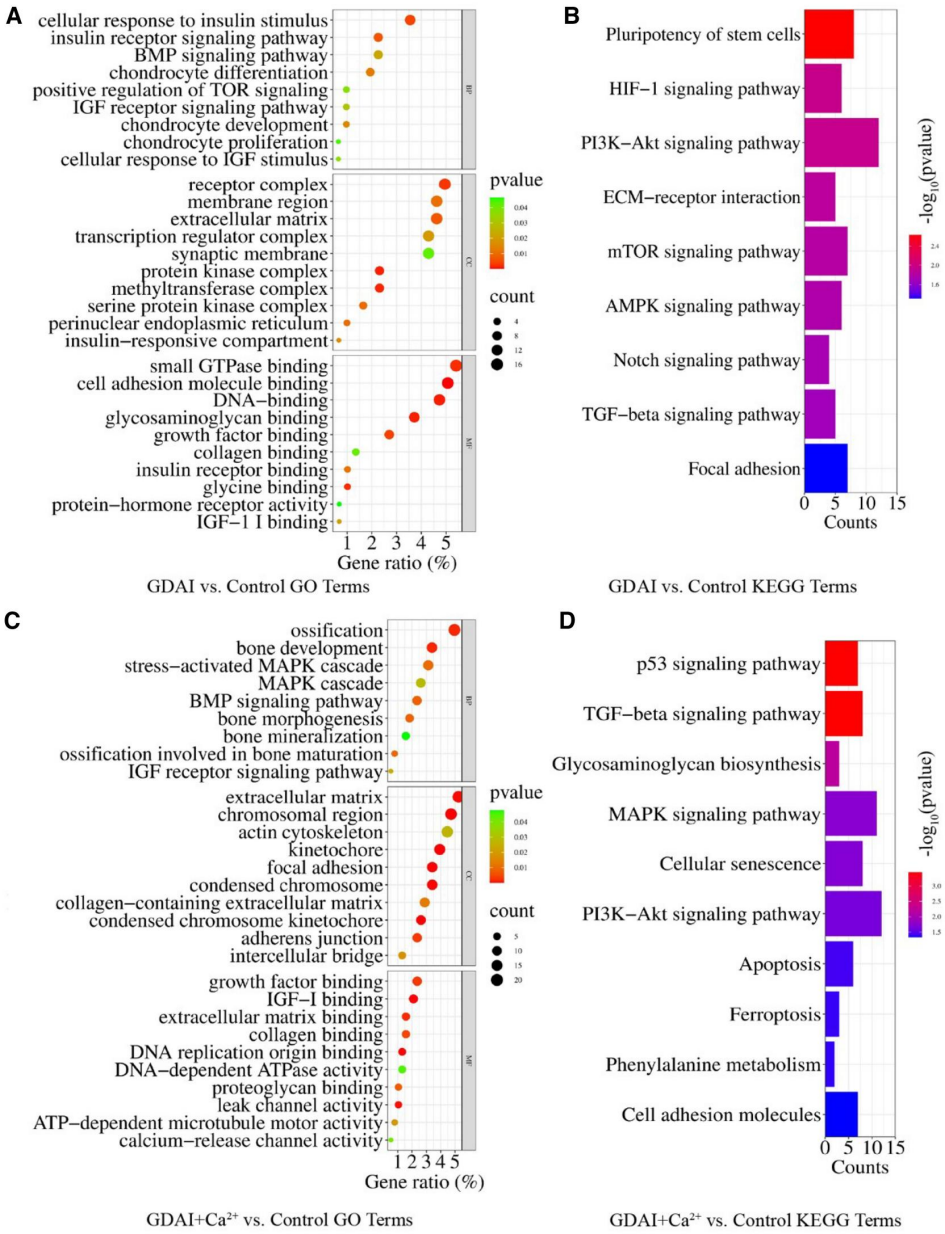
Transcriptome analysis of hydrogels function on BMSCs. The top up enrichment GO terms in the GDAI vs. control hydrogel group (**A**), the top up enrichment KEGG pathways in the GDAI vs. control group (**B**), the top up enrichment GO terms in the GDAI+Ca^2+^ vs. control hydrogel group (**C**), the top up enrichment KEGG pathways in the GDAI+ Ca^2+^ vs. control group (**D**).

The GO enrichment was conducted to examine the functional implications of the DEGs in the GDAI vs. control group (Figure 6A). Regarding biological processes (BP), the insulin and IGF-1 receptor signaling pathways were upregulated in the cellular response to insulin stimulus. Regarding cellular components (CC), notable features included the protein kinase complex, insulin-responsive compartment and transcription regulator complex. These findings suggested that the material groups could release IGF-1, promoting chondrocyte proliferation, development and differentiation. Furthermore, the KEGG enrichment was done to elucidate the functional pathways associated with the DEGs in the GDAI vs. control group (Figure 6B). There were significant differences in the pluripotency of stem cells, implying that stem cells could undergo directed differentiation in the presence of growth factors. The activation of the PI3K-Akt-mTOR pathway indicated that IGF-1 exerted its effects on chondrocytes via this pathway. The activation of the AMPK pathway, Notch pathway and TGF-β pathway was because the “PI3K-Akt” signaling pathway was downstream of multiple signaling processes and controlled various cellular functions, consistent with recent studies [32, 33], The hypoxia inducible factor-1 (HIF-1) signaling pathway, a critical microenvironmental factor in regulating chondrocyte differentiation [34, 35], was significantly upregulated.

Figure 6C shows the GO enrichment results of the GDAI+Ca^2+^ vs. the control group. Regarding BP, an upregulation was observed in bone development, ossification, mineralization and bone maturation. In the CC category, the focus was on the ECM and chromosomal regions. The MF results indicated IGF-1 binding, activation of the calcium pathway, ATP activity, DNA binding, etc. The KEGG pathway (Figure 6D) revealed the activation of several pathways, including the p53 pathway, TGF-β pathway, mitogen-activated protein kinase (MAPK) pathway and PI3K-Akt pathway. These findings suggested that the calcium ions could promote the directed differentiation of BMSCs into osteoblasts. However, the IGF-1 receptor pathway indicated that even with the enhancement of material strength by calcium ions, the internal IGF-1 could still be released and function.

String database was used to perform subsequent analysis of PPI, which indicated a potential association of differentially expressed proteins with specific signaling pathways. In the GDAI+Ca^2+^ vs. control group, Map3k5 was found to be associated with the MAPK signaling pathway, and Pbk was associated with the PI3K-Akt signaling pathway, confirming the results from the GO and KEGG enrichment analyses (Figure 5D). In the GDAI vs. control group, IGF-1R was associated with the PI3K-Akt signaling pathway, Crebbp was related to the TGF-β signaling pathway, and Notch2 with bone remodeling and homeostasis (Figure 5E). WB results demonstrated increased levels of p-Akt and p-PI3K on day 7 (Figure 5F), verifying the activation of the relevant signal pathway, which aligned with the GO and KEGG enrichment findings.

### Evaluation of growth plates regeneration efficacy *in vivo*

The different hydrogel groups were implanted into rat models with proximal tibial growth plate injury to assess the regenerative capability in repairing the growth plate *in vivo*.

**Figure 7.**
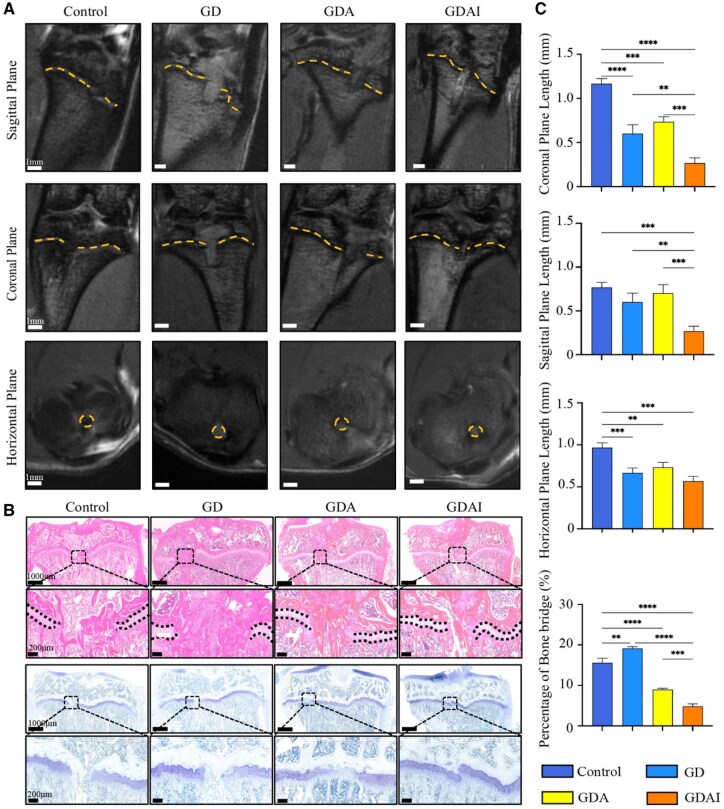
Hydrogels accelerate growth plate healing *in vivo*. New bone formation in the control, GD, GDA and GDAI hydrogel groups at 8 weeks observed by MRI (**A**), HE and TB (**B**) staining of the growth plate in the control, GD, GDA and GDAI groups. Quantification of bone bridge length and percentage of bone bridge, measured by ImageJ (**C**). The asterisk (*) represents a significant difference (*P < 0.05, **P < 0.01, ***P < 0.001, ****P < 0.0001).

At 8 weeks post-surgery, the growth plate repair rate was evaluated using MRI. In the coronal plane, the control group exhibited the largest bone bridge, followed by the GD and GDA groups, respectively, while the GDAI group displayed the smallest residual bone bridge (Figure 7A). Statistically significant differences were observed among all groups. In the sagittal plane, there was an insignificant difference between the bone bridge length in the control, GD and GDA groups, but all were significantly larger than in the GDAI group. Furthermore, the control group exhibited the largest bone bridge in the horizontal plane, significantly larger than those in the other groups. Conversely, the GDAI group had the smallest bone bridge length. These results indicated that the GDAI hydrogel effectively reduced the occurrence of bone bridges following growth plate injury *in vivo*.

The results of HE staining showed that the control group displayed the largest bone bridges, followed by the GD group, while the GDAI group exhibited the smallest bone bridges. Subsequently, TB staining results revealed evident bone bridge formation in the control group. On the contrary, other groups exhibited visible regeneration and connection of the growth plate. The GDA group showed partial connection, albeit with lighter staining and disordered chondrocytes. The growth plate at the broken end in the GDAI group exhibited substantial reconnection, with neatly arranged chondrocytes among themselves (Figure 7B). The percentage of bone bridges was quantified using ImageJ software (Figure 7C). The GDAI group demonstrated the smallest bone bridges, with a statistically significant difference, compared with other groups.

### DEGs comparison revealed a hydrogel characteristic *in vivo*

**Figure 8.**
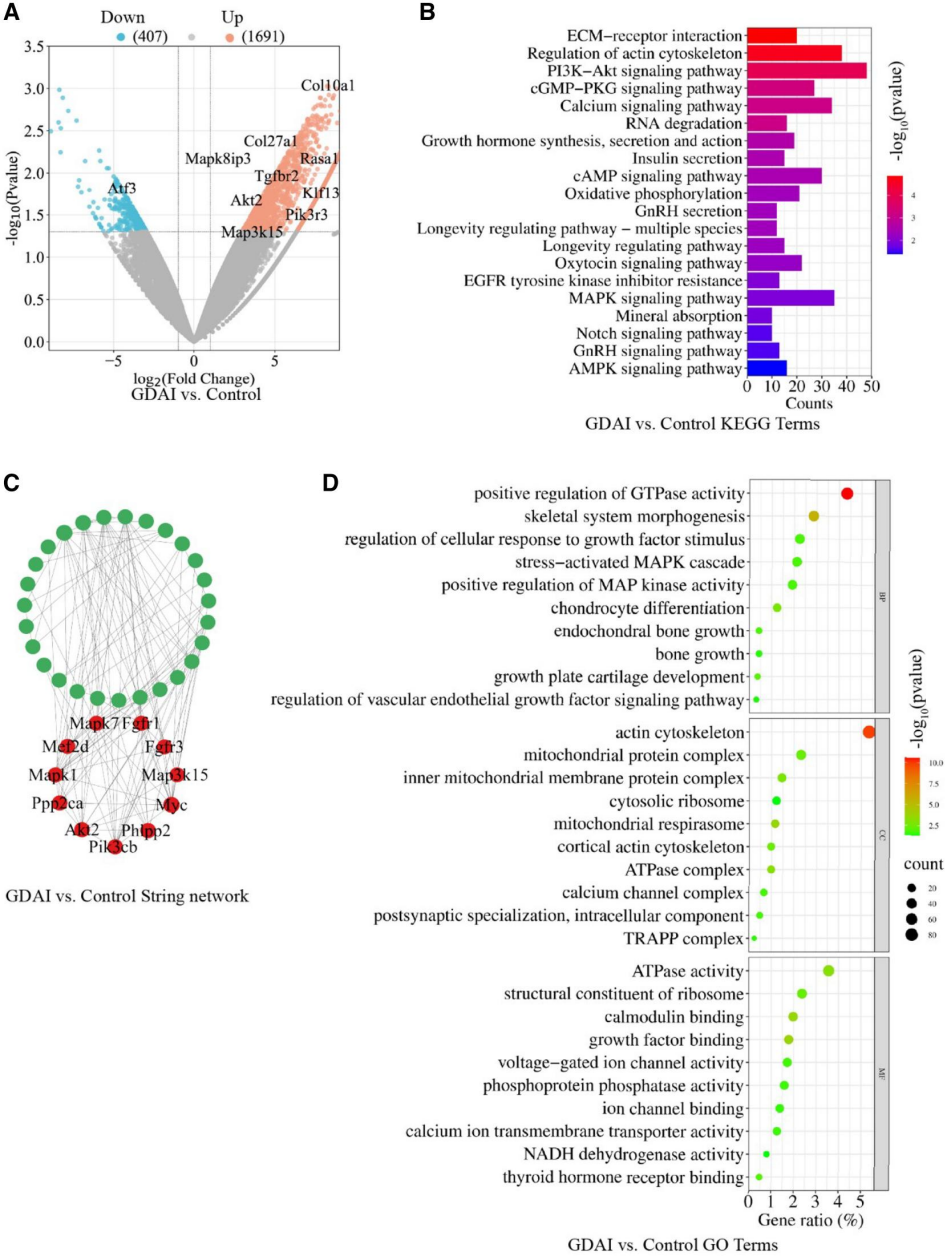
Transcriptome analysis of hydrogels function *in vivo*. Volcano plot showing the number of DEGs in the control and GDAI groups (**A**); the top up enrichment KEGG pathways in the control and GDAI groups (**B**), the top upregulated PPI analysis of the GDAI group (**C**), the top up enrichment GO terms in the GDAI and control groups (**D**). DEGs were presented with FoldChange > 2 or FoldChange < 0.5.

The RNA-seq assays were conducted on the control and GDAI groups to gain further insights into the mechanism of the hydrogels *in vivo*. Figure 8A shows the DEGs between the two groups, wherein genes related to cartilage regeneration were significantly upregulated in the GDAI group, such as col10a1, col27a1, klf13, akt2, pik3r3, tgfbr2 and rasa1.

KEGG analysis (Figure 8B) demonstrated that the significantly activated pathways included the PI3K-Akt signaling pathway, calcium pathway, growth hormone-related pathway, MAPK pathway, AMPK pathway, Notch pathway, oxidative phosphorylation and mineral deposition pathway. Furthermore, the hydrogels injected into the injury site could seal the wound, create a hypoxic microenvironment and promote cartilage regeneration through the HiF-1α signaling pathway. Moreover, string analysis further supported the activation of the PI3K-Akt and related signaling pathways (Figure 8C). GO enrichment revealed that the DEGs primarily influenced BP related to the skeletal system, encompassing cartilage differentiation, endogenous cartilage growth, bone growth, growth plate development and activation of the MAPK and vascular endothelial growth factor (VEGF) signaling pathways. Regarding CC, the DEGs were associated with intracellular skeletons, mitochondria, ribosomes and calcium channel complexes. The MF analysis indicated the activation of ATP, ribosome assembly, calmodulin binding, growth factor binding, ion channel binding and other functions. The activated pathways involved ATP activation, ribosome assembly and calmodulin binding (Figure 8D). These findings were consistent with the pathways activated in cellular experiments.

**Figure 9.**
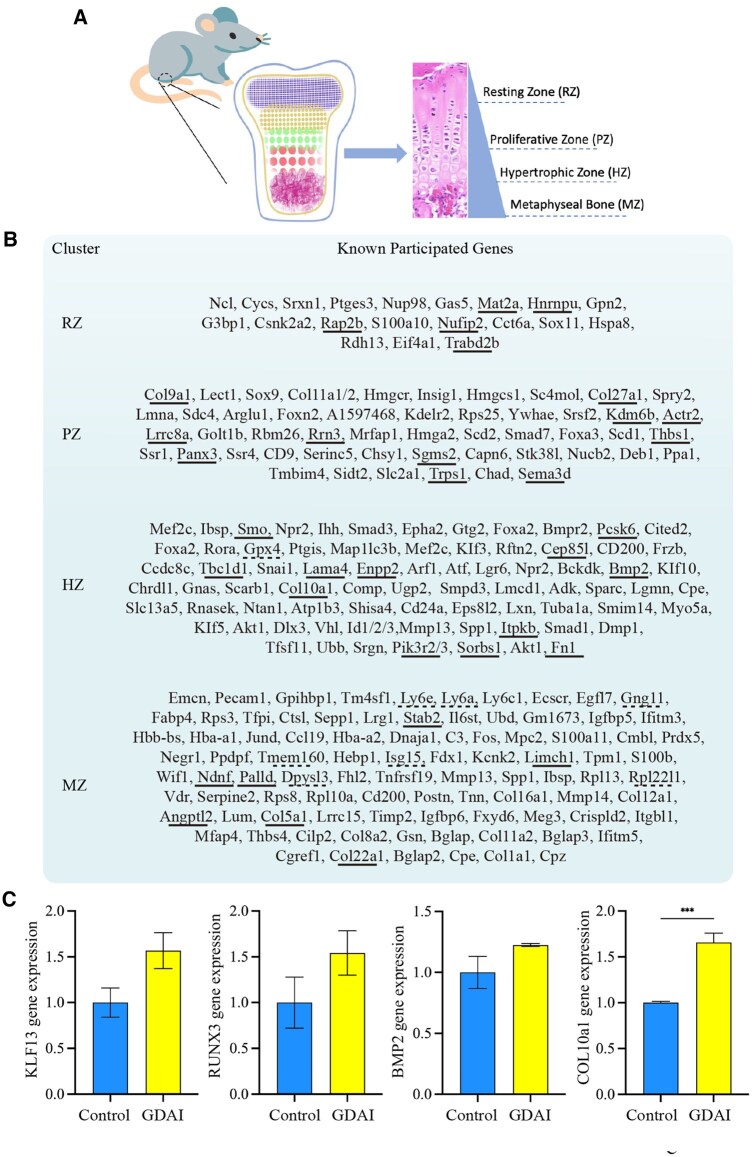
Comparison of known growth plate genes (adapted from Li et al., 2016 [36]) with DEGs results. Schematic diagram of the process (**A**), previously reported or experimentally validated genes that participate in GP or cartilage development in each cluster are listed under “known participated genes’’, the upregulated DEGs were marked in solid lines and the downregulated DEGs were marked in dashed lines (**B**), key markers of growth plate were validated by RT-qPCR (**C**) (***P < 0.001).

The primary genes expressed in each layer of the growth plate were reported in previous studies [36, 37] and compared with our RNA-seq results (Figure 9A). The red indicated significantly upregulated DEGs, and the blue indicated significantly downregulated DEGs (Figure 9B). The results showed that most genes were upregulated from the resting zone to the hypertrophic zone, while more genes were downregulated in the calcified zone. This suggested that the implanted material could effectively activate gene expression in the growth plate’s resting, proliferative and hypertrophic layers while inhibiting the expression of osteogenic genes in the calcified layer. According to the literature, KLF13 and RUNX3 are known to be the critical markers involved in the regulation of growth plate development [36], BMP2 is significantly expressed in the hypertrophic zone of the growth plate [37] and collagen, type X, alpha 1 (COL10A1) is expressed explicitly in the hypertrophic zone and serves as a landmark marker for growth plate detection [38]. The RT-qPCR results demonstrated significantly increased gene expression (KLF13, RUNX3, BMP2 and COL10A1) in the GDAI group compared to the control group, indicating that the experimental group could better promote the regenerative repair of growth plate cartilage (Figure 9C).

## Discussion

The growth plate in children is a distinctive cartilaginous tissue between the epiphysis and diaphysis of long bones. A regular longitudinal growth is ensured by endochondral ossification, which begins during fetal development and continues till the end of puberty [39]. Due to its vulnerability, growth plate injuries constitute one-third of fractures in children. These injuries have been shown to result in skeletal deformities, limb length discrepancies, and joint stiffness, significantly impacting children's physical health and daily activities. Current treatments primarily involve the removal of bone bridges formed after damage to the growth plate and their replacement with fat or bone wax. However, these treatments are invasive and lack stability. Although growth hormone has shown potential in stimulating growth plate activity through IGF-1, its application in growth plate injury has not been successful. Any injury to the growth plate disrupts the local microenvironment, impeding the transmission of growth signals, suppressing chondrogenic genes and overexpressing osteogenic genes. Therefore, developing alternative biomaterials based on the structure and physiological function of the growth plate represents a more promising approach.

Bone bridge formation following growth plate injury primarily involves intrachondral osteogenesis [3]. Therefore, the first task to stop this process involves inhibiting the hematoma formation. Previous literature has reported the use of adhesive hydrogels for hemostasis. Wei et al. [40] developed hemostatic sponges based on chitosan and oxidized cellulose, demonstrating their intrinsic hemostatic effect. Huang et al. [41] synthesized aldehyde-based nano-bacterial cellulose hydrogels through Schiff base reactions with catechol-grafted chitosan, showing promising hemostatic properties both *in vitro* and *in vivo*. The hydrogels used in this study were photo-crosslinked *in situ* under UV light, offering rapid gelation and excellent biomechanical properties. Consequently, the hydrogel was applied at the injury site, sealing it, and preventing blood infiltration, inflammatory cell infiltration and hematoma formation. The growth plate cartilage is present in a hypoxic microenvironment, which promotes chondrocyte differentiation, cartilage synthesis and proliferation.

The growth plate is a specialized structure between the epiphysis and metaphysis and has a “hamburger” shape. This unique configuration protects the internal cartilage structure, facilitating proper growth and elongation. Previous research has demonstrated the significant influence of ECM on cell proliferation and lineage commitment [42]. Gong et al. [43] reported that the double-network hydrogel, constructed by combining two networks with different structures and densities, could effectively relax the locally applied stress and dissipate the crack energy, thereby improving its mechanical strength. Lin et al. [44] reported that designing anti-fatigue-fracture hydrogels requires making the fatigue crack encounter and fracture objects with energies per unit area much higher than that for fracturing a single layer of polymer chains. Inspired by that, a high-toughness adaptive dual-crosslinked hydrogel was prepared by forming Schiff base bonds between the amino group of GelMA, the aldehyde group of DBNC and Alg. Thus, the hydrogel employed in this study could dynamically form varying degrees of matrix stiffness *in vivo*, tailored to match the pathological conditions of the growth plate following injury.

Furthermore, the hydrogel could recruit calcium ions from the cancellous bone at contact sites with the upper and lower aspects of the growth plate, facilitating their binding to the hydrogel. This interaction resulted in a dense hydrogel structure at the bone interface, representing a rigid matrix, while the central portion maintained a soft matrix state with internal hypoxia. The recruited calcium ions played a crucial role in the normal development of growth plate structures and longitudinal bone growth, participating in the proliferation and differentiation of growth plate chondrocytes [45]. The study results indicated that calcium ions could enhance the osteogenic differentiation of BMSCs and specifically activate the p53, TGF-β and MAPK signaling pathways. On the contrary, when calcium ions coexisted with IGF-1, the expression of osteogenic genes decreased, while chondrogenic genes were significantly upregulated. This distinct regulation of osteogenic and chondrogenic functions was effectively aligned with the expected growth process of children’s growth plates, providing energy for bone growth during childhood.

The ECM of the growth plate comprises a natural cartilaginous structure and contains various growth factors that promote cartilage proliferation, differentiation and vascular regeneration [46]. The GF/IGF-1 axis plays a critical role in maintaining the integrity of the cartilage structure. The GF/IGF-1 axis has been demonstrated to stimulate the proliferation and differentiation of growth plate cartilage, exerting systemic and local effects [47, 48]. Previous studies have shown that IGF-1-loaded polymers enhance the proliferation and chondrogenic differentiation of BMSCs for repairing articular cartilage. Some examples include Poly(ε-caprolactone) (PCL) scaffolds incorporating poly(lactic-co-glycolic acid) (PLGA) nanoparticles [49], polyethylene glycol-modified Poly-amidoamine (PAMAM) dendrimer nanocarriers [50] and Alg hydrogels carrying recombinant adeno-associated virus (rAAV) [51]. In this study, drug release assays confirmed that the hydrogels developed sustained the slow release of IGF-1 growth factor, targeting the cartilage differentiation ability of BMSCs. Here, the IGF-1-containing hydrogels downregulated the expression of relevant osteogenic genes and upregulated the expression of chondrogenic genes. Furthermore, RNA-seq assays indicated the activation of the PI3K-Akt pathway, suggesting that IGF-1 exerted its effects on chondrocytes via this pathway. The AMPK, Notch and TGF-β pathways activated because the “PI3K-Akt” signaling pathway functioned downstream of multiple signaling processes and controlled various cellular functions [32, 33].

Thus, this novel hydrogel material exhibited soft matrix characteristics without calcium ions, facilitating the chondrogenic differentiation of BMSCs. Adding IGF-1 further enhanced this effect by activating relevant chondrogenic differentiation signaling pathways in BMSCs, such as the PI3K-Akt signaling and TGF-β signaling pathways [52]. When calcium ions were present, the hydrogel gradually transformed into a rigid matrix by recruiting calcium ions from the surrounding tissues. This transformation promoted cytoskeletal organization and microtubule formation in BMSCs. However, the hydrogel material maintained the ability to release IGF-1, thereby reducing the expression of relevant osteogenic genes and preventing bone bridge formation.

In the future, biomaterials are expected to make significant advancements in growth plate injury repair. For example, bone tissue engineering scaffolds incorporating rigid 3D-printed outer layers and soft hydrogels infused inside [53] can mimic the mechanical properties and ECM of the growth plate. Composite scaffolds with smart, bioactive materials that regulate immune responses, angiogenesis and cartilage differentiation [54] offer a novel and promising approach to the treatment of growth plate injuries.

## Conclusion

In summary, a novel high-toughness adaptive dual-crosslinked hydrogel was developed that emulated the unique layered structure of the growth plate. This hydrogel was created by combining DBNC, GelMA and Alg materials via Schiff base bonding and ionic bonding electrostatic attraction while incorporating IGF-1 to promote overexpression in the local microenvironment. The primary aim was to reshape the structure and functionality of the growth plate to facilitate bone growth. The hydrogel demonstrated rapid sealing properties, in turn, closing the damaged area of the growth plate and establishing an appropriate hypoxic microenvironment to support cartilage regeneration. The hydrogel continuously extracted calcium ions from the surrounding cancellous bone and blood, nourishing the regenerating growth plate and reinforcing the structure of the hydrogel. The localized release of IGF-1 from the hydrogel activated the downstream PI3K-Akt signaling pathway, which was crucial in promoting the regeneration and repair of growth plate cartilage. Thus, this innovative hydrogel presents a promising approach for regenerative applications to treat diseases associated with pediatric growth plate injuries. It offers a new avenue for addressing these conditions and holds the potential for advancing therapeutic interventions in this area.

## Supplementary Material

rbaf004_Supplementary_Data

## Data Availability

The raw/processed data can be obtained from the authors on request.

## References

[1] Shaw N , Erickson C, Bryant SJ, Ferguson VL, Krebs MD, Hadley-Miller N, Payne KA. Regenerative medicine approaches for the treatment of pediatric physeal injuries. Tissue Eng Part B Rev 2018;24:85–97. 28830302 10.1089/ten.teb.2017.0274 PMC5905866

[2] Salhotra A , Shah HN, Levi B, Longaker MT. Mechanisms of bone development and repair. Nat Rev Mol Cell Biol 2020;21:696–711. 32901139 10.1038/s41580-020-00279-w PMC7699981

[3] Zhou FH , Foster BK, Sander G, Xian CJ. Expression of proinflammatory cytokines and growth factors at the injured growth plate cartilage in young rats. Bone 2004;35:1307–15. 15589211 10.1016/j.bone.2004.09.014

[4] Macsai CE , Hopwood B, Chung R, Foster BK, Xian CJ. Structural and molecular analyses of bone bridge formation within the growth plate injury site and cartilage degeneration at the adjacent uninjured area. Bone 2011;49:904–12. 21807132 10.1016/j.bone.2011.07.024

[5] Ngo TQ , Scherer MA, Zhou FH, Foster BK, Xian CJ. Expression of bone morphogenic proteins and receptors at the injured growth plate cartilage in young rats. J Histochem Cytochem 2006;54:945–54. 16651391 10.1369/jhc.6A6939.2006

[6] Fischerauer E , Heidari N, Neumayer B, Deutsch A, Weinberg AM. The spatial and temporal expression of VEGF and its receptors 1 and 2 in post-traumatic bone bridge formation of the growth plate. J Mol Histol 2011;42:513–22. 21928073 10.1007/s10735-011-9359-x

[7] Sakata R , Iwakura T, Reddi AH. Regeneration of articular cartilage surface: morphogens, cells, and extracellular matrix scaffolds. Tissue Eng Part B Rev 2015;21:461–73. 25951707 10.1089/ten.TEB.2014.0661

[8] Arasapam G , Scherer M, Cool JC, Foster BK, Xian CJ. Roles of COX-2 and iNOS in the bony repair of the injured growth plate cartilage. J Cell Biochem 2006;99:450–61. 16619262 10.1002/jcb.20905

[9] Xian CJ , Zhou FH, McCarty RC, Foster BK. Intramembranous ossification mechanism for bone bridge formation at the growth plate cartilage injury site. J Orthop Res 2004;22:417–26. 15013105 10.1016/j.orthres.2003.08.003

[10] Mahran YF , Badr AM, Aldosari A, Bin-Zaid R, Alotaibi HN. Carvacrol and thymol modulate the Cross-Talk between TNF-α and IGF-1 signaling in Radiotherapy-Induced ovarian failure. Oxid Med Cell Longev 2019;2019:3173745. 31531182 10.1155/2019/3173745 PMC6721489

[11] McCarty RC , Xian CJ, Gronthos S, Zannettino AC, Foster BK. Application of autologous bone marrow derived mesenchymal stem cells to an ovine model of growth plate cartilage injury. Open Orthop J 2010;4:204–10. 20721323 10.2174/1874325001004010204 PMC2923344

[12] Guan P , Liu C, Xie D, Mao S, Ji Y, Lin Y, Chen Z, Wang Q, Fan L, Sun Y. Exosome-loaded extracellular matrix-mimic hydrogel with anti-inflammatory property facilitates/promotes growth plate injury repair. Bioact Mater 2022;10:145–58. 34901536 10.1016/j.bioactmat.2021.09.010 PMC8637006

[13] Guo J , Luo Z, Wang F, Gu H, Li M. Responsive hydrogel microfibers for biomedical engineering. Smart Med 2022;1:e20220003. 39188750 10.1002/SMMD.20220003 PMC11235791

[14] Zhao X. Multi-scale multi-mechanism design of tough hydrogels: building dissipation into stretchy networks. Soft Matter 2014;10:672–87. 24834901 10.1039/C3SM52272E PMC4040255

[15] Yang S , Wang F, Han H, Santos HA, Zhang Y, Zhang H, Wei J, Cai Z. Fabricated technology of biomedical micro-nano hydrogel. Biomedical Technology 2023;2:31–48.

[16] Zhang H , Xu D, Zhang Y, Li M, Chai R. Silk fibroin hydrogels for biomedical applications. Smart Med 2022;1:e20220011. 39188746 10.1002/SMMD.20220011 PMC11235963

[17] Zhang Z , Bao L, Qian C, Furtado M, Li H, Guo S, Zheng Y, Fu D, Dong K, Cui W, Wang D. The high-strength and toughness janus bionic periosteum matching bone development and growth in children. Compos B Eng 2023;256:110642.

[18] Zhang W , Wang XC, Li XY, Zhang LL, Jiang F. A 3D porous microsphere with multistage structure and component based on bacterial cellulose and collagen for bone tissue engineering. Carbohydr Polym 2020;236:116043. 32172857 10.1016/j.carbpol.2020.116043

[19] Liu W , Madry H, Cucchiarini M. Application of alginate hydrogels for next-generation articular cartilage regeneration. Int J Mol Sci 2022;23:10.3390/ijms23031147 PMC8835677 35163071

[20] Matsumoto T , Tsurumoto T, Goldring MB, Shindo H. Differential effects of IGF-binding proteins, IGFBP-3 and IGFBP-5, on IGF-I action and binding to cell membranes of immortalized human chondrocytes. J Endocrinol 2000;166:29–37. 10856880 10.1677/joe.0.1660029

[21] Yang R , Li G, Zhuang C, Yu P, Ye T, Zhang Y, Shang P, Huang J, Cai M, Wang L, Cui W, Deng L. Gradient bimetallic ion-based hydrogels for tissue microstructure reconstruction of tendon-to-bone insertion. Sci Adv 2021;7:eabg3816. 10.1126/sciadv.abg3816 PMC8221628 34162547

[22] Erickson CB , Shaw N, Hadley-Miller N, Riederer MS, Krebs MD, Payne KA. A rat tibial growth plate injury model to characterize repair mechanisms and evaluate growth plate regeneration strategies. J Vis Exp 2017;125:55571. 10.3791/55571 PMC5608538 28715376

[23] Guo JF , Jourdian GW, MacCallum DK. Culture and growth characteristics of chondrocytes encapsulated in alginate beads. Connect Tissue Res 1989;19:277–97. 2805684 10.3109/03008208909043901

[24] Cohen SB , Meirisch CM, Wilson HA, Diduch DR. The use of absorbable co-polymer pads with alginate and cells for articular cartilage repair in rabbits. Biomaterials 2003;24:2653–60. 12726719 10.1016/s0142-9612(03)00058-9

[25] Madry H , Cucchiarini M, Stein U, Remberger K, Menger MD, Kohn D, Trippel SB. Sustained transgene expression in cartilage defects in vivo after transplantation of articular chondrocytes modified by lipid-mediated gene transfer in a gel suspension delivery system. J Gene Med 2003;5:502–9. 12797115 10.1002/jgm.368

[26] Madry H , Kaul G, Cucchiarini M, Stein U, Zurakowski D, Remberger K, Menger MD, Kohn D, Trippel SB. Enhanced repair of articular cartilage defects in vivo by transplanted chondrocytes overexpressing insulin-like growth factor I (IGF-I). Gene Ther 2005;12:1171–9. 15815701 10.1038/sj.gt.3302515

[27] Chuang CY , Shahin K, Lord MS, Melrose J, Doran PM, Whitelock JM. The cartilage matrix molecule components produced by human foetal cartilage rudiment cells within scaffolds and the role of exogenous growth factors. Biomaterials 2012;33:4078–88. 22391264 10.1016/j.biomaterials.2012.02.032

[28] Lin F , Li Y, Cui W. Injectable hydrogel microspheres in cartilage repair. Biomedical Technology 2023;1:18–29.

[29] Kadri R , Elkhoury K, Ben Messaoud G, Kahn C, Tamayol Ali, Mano JF, Arab-Tehrany E, Sánchez-González L. Physicochemical interactions in nanofunctionalized alginate/gelma IPN hydrogels. Nanomaterials **2021**;11:2256. Doi: 10.3390/nano11092256. 34578572 PMC8465058

[30] Khanjani P , Ristolainen M, Kosonen H, Virtanen P, Ceccherini S, Maloney T, Vuorinen T. Time-triggered calcium ion bridging in preparation of films of oxidized microfibrillated cellulose and pulp. Carbohydr Polym 2019;218:63–7. 31221344 10.1016/j.carbpol.2019.04.060

[31] Busuioc C , Isopencu G, Banciu A, Banciu D-D, Oprea O, Mocanu A, Deleanu I, Zăuleţ M, Popescu L, Tănăsuică R, Vasilescu M, Stoica-Guzun A. Bacterial cellulose hybrid composites with calcium phosphate for bone tissue regeneration. IJMS **2022**;23:16180. Doi: 10.3390/ijms232416180. 36555821 PMC9784094

[32] Li J , Zhang B, Liu WX, Lu K, Pan H, Wang T, Oh CD, Yi D, Huang J, Zhao L, Ning G, Xing C, Xiao G, Liu-Bryan R, Feng S, Chen D. Metformin limits osteoarthritis development and progression through activation of AMPK signalling. Ann Rheum Dis 2020;79:635–45. 32156705 10.1136/annrheumdis-2019-216713 PMC7213329

[33] Wang Q , Zhou C, Li X, Cai L, Zou J, Zhang D, Xie J, Lai W. TGF-β1 promotes gap junctions formation in chondrocytes via Smad3/Smad4 signalling. Cell Prolif 2019;52:e12544. 30444057 10.1111/cpr.12544 PMC6495951

[34] Stegen S , Laperre K, Eelen G, Rinaldi G, Fraisl P, Torrekens S, Van Looveren R, Loopmans S, Bultynck G, Vinckier S, Meersman F, Maxwell PH, Rai J, Weis M, Eyre DR, Ghesquière B, Fendt SM, Carmeliet P, Carmeliet G. HIF-1α metabolically controls collagen synthesis and modification in chondrocytes. Nature 2019;565:511–5. 30651640 10.1038/s41586-019-0874-3 PMC7195049

[35] Yao Q , Khan MP, Merceron C, LaGory EL, Tata Z, Mangiavini L, Hu J, Vemulapalli K, Chandel NS, Giaccia AJ, Schipani E. Suppressing mitochondrial respiration is critical for hypoxia tolerance in the fetal growth plate. Dev Cell 2019;49:748–63.e7. 31105007 10.1016/j.devcel.2019.04.029 PMC7255488

[36] Li J , Luo H, Wang R, Lang J, Zhu S, Zhang Z, Fang J, Qu K, Lin Y, Long H, Yao Y, Tian G, Wu Q. Systematic reconstruction of molecular Cascades regulating GP development using single-cell RNA-Seq. Cell Rep 2016;15:1467–80. 27160914 10.1016/j.celrep.2016.04.043

[37] Minina E , Wenzel HM, Kreschel C, Karp S, Gaffield W, McMahon AP, Vortkamp A. BMP and ihh/PTHrP signaling interact to coordinate chondrocyte proliferation and differentiation. Development 2001;128:4523–34. 11714677 10.1242/dev.128.22.4523

[38] Myllyharju J. Extracellular matrix and developing growth plate. Curr Osteoporos Rep 2014;12:439–45. 25212565 10.1007/s11914-014-0232-1

[39] Hosseinzadeh P , Milbrandt T. The normal and fractured physis: an anatomic and physiologic overview. J Pediatr Orthop B 2016;25:385–92. 26523532 10.1097/BPB.0000000000000245

[40] Wei X , Ding S, Liu S, Yang K, Cai J, Li F, Wang C, Lin S, Tian F. Polysaccharides-modified chitosan as improved and rapid hemostasis foam sponges. Carbohydr Polym 2021;264:118028. 33910719 10.1016/j.carbpol.2021.118028

[41] Huang W , Cheng S, Wang X, Zhang Y, Chen L, Zhang L. Noncompressible hemostasis and bone regeneration induced by an absorbable bioadhesive self-healing hydrogel. Adv Funct Materials 2021;31:2009189.

[42] Liu N , Zhou M, Zhang Q, Yong L, Zhang T, Tian T, Ma Q, Lin S, Zhu B, Cai X. Effect of substrate stiffness on proliferation and differentiation of periodontal ligament stem cells. Cell Prolif 2018;51:e12478. 30039894 10.1111/cpr.12478 PMC6528973

[43] Gong JP , Katsuyama Y, Kurokawa T, Osada Y. Double-Network hydrogels with extremely high mechanical strength. Adv Mater 2003;15:1155–8.

[44] Lin S , Liu X, Liu J, Yuk H, Loh HC, Parada GA, Settens C, Song J, Masic A, McKinley GH, Zhao X. Anti-fatigue-fracture hydrogels. Sci Adv 2019;5:eaau8528. 30746464 10.1126/sciadv.aau8528 PMC6357728

[45] Mancilla EE , Galindo M, Fertilio B, Herrera M, Salas K, Gatica H, Goecke A. L-type calcium channels in growth plate chondrocytes participate in endochondral ossification. J Cell Biochem 2007;101:389–98. 17243114 10.1002/jcb.21183

[46] Horton ER , Vallmajo-Martin Q, Martin I, Snedeker JG, Ehrbar M, Blache U. Extracellular matrix production by mesenchymal stromal cells in hydrogels facilitates cell spreading and is inhibited by FGF-2. Adv Healthc Mater 2020;9:e1901669. 32129003 10.1002/adhm.201901669

[47] Dixit M , Poudel SB, Yakar S. Effects of GH/IGF axis on bone and cartilage. Mol Cell Endocrinol 2021;519:111052. 33068640 10.1016/j.mce.2020.111052 PMC7736189

[48] Abbaspour A , Takata S, Matsui Y, Katoh S, Takahashi M, Yasui N. Continuous infusion of insulin-like growth factor-I into the epiphysis of the tibia. Int Orthop 2008;32:395–402. 17431620 10.1007/s00264-007-0336-7 PMC2323423

[49] Wei P , Xu Y, Gu Y, Yao Q, Li J, Wang L. IGF-1-releasing PLGA nanoparticles modified 3D printed PCL scaffolds for cartilage tissue engineering. Drug Deliv 2020;27:1106–14. 32715779 10.1080/10717544.2020.1797239 PMC7470157

[50] Geiger BC , Wang S, Padera RF Jr., Grodzinsky AJ, Hammond PT. Cartilage-penetrating nanocarriers improve delivery and efficacy of growth factor treatment of osteoarthritis. Sci Transl Med 2018;10:eaat8800. 10.1126/scitranslmed.aat8800 PMC8103617 30487252

[51] Maihöfer J , Madry H, Rey-Rico A, Venkatesan JK, Goebel L, Schmitt G, Speicher-Mentges S, Cai X, Meng W, Zurakowski D, Menger MD, Laschke MW, Cucchiarini M. Hydrogel-Guided, rAAV-Mediated IGF-I overexpression enables Long-Term cartilage repair and protection against perifocal osteoarthritis in a Large-Animal Full-Thickness chondral defect model at one year In vivo. Adv Mater 2021;33:e2008451. 33734514 10.1002/adma.202008451 PMC11468525

[52] Klampfleuthner FAM , Lotz B, Renkawitz T, Richter W, Diederichs S. Stage-dependent activity and pro-chondrogenic function of PI3K/AKT DURING cartilage neogenesis from mesenchymal stromal cells. Cells 2022;11:2965. Doi: 10.3390/cells11192965. 36230927 PMC9563299

[53] Zhang J , Tong D, Song H, Ruan R, Sun Y, Lin Y, Wang J, Hou L, Dai J, Ding J, Yang H. Osteoimmunity-regulating biomimetically hierarchical scaffold for augmented bone regeneration. Adv Mater 2022;34:e2202044. 35785450 10.1002/adma.202202044

[54] Liu Z , Zhang J, Fu C, Ding J. Osteoimmunity-regulating biomaterials promote bone regeneration. Asian J Pharm Sci 2023;18:100774. 36751654 10.1016/j.ajps.2023.100774 PMC9894904

