# Supplementary material for: Up IGF-I via high-toughness adaptive hydrogels for remodeling growth plate of children
Source: Regen Biomater. 2025 Jan 23;12:rbaf004. doi: 10.1093/rb/rbaf004 (PMC11897792; doi:10.1093/rb/rbaf004)
Supplement: rbaf004_Supplementary_Data [file rbaf004_supplementary_data.doc]

Supporting Information

Up IGF-I via High-toughness Adaptive Hydrogels for Remodeling Growth Plate of Children

Zhiqiang Zhang^1^, Haodong Li^1^, Manning Qian^1^, Yiming Zheng^1^, Luhan Bao^2*^, Wenguo Cui^2*^, Dahui Wang^1*^

^1^Department of Orthopedics, National Children’s Medical Center & Children’s Hospital of Fudan University, 399 Wan Yuan Road, Shanghai, 201102, China

^2^Department of Orthopaedics, Shanghai Key Laboratory for Prevention and Treatment of Bone and Joint Diseases, Shanghai Institute of Traumatology and Orthopaedics, Ruijin Hospital, Shanghai Jiao Tong University School of Medicine, 197 Ruijin 2nd Road, Shanghai 200025, P. R. China.

* Corresponding author: Luhan Bao: baoluhancome@sina.cn, Wenguo Cui [wgcui90@hotmail.com](mailto:wgcui90@hotmail.com), Dahui Wang: [wangdahui@fudan.edu.cn](mailto:wangdahui@fudan.edu.cn)


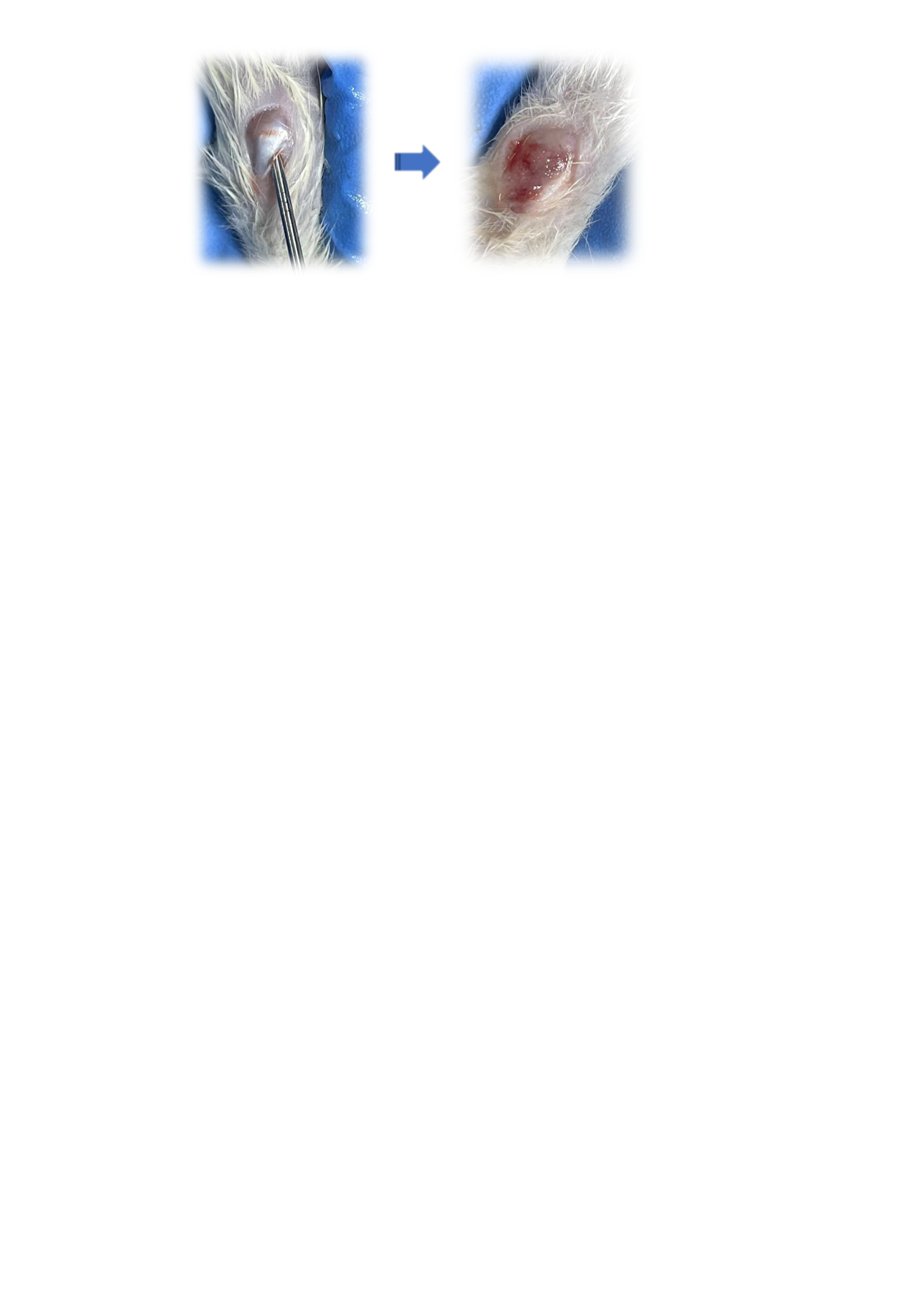


Figure S1. Schematic diagram of the surgical procedure.


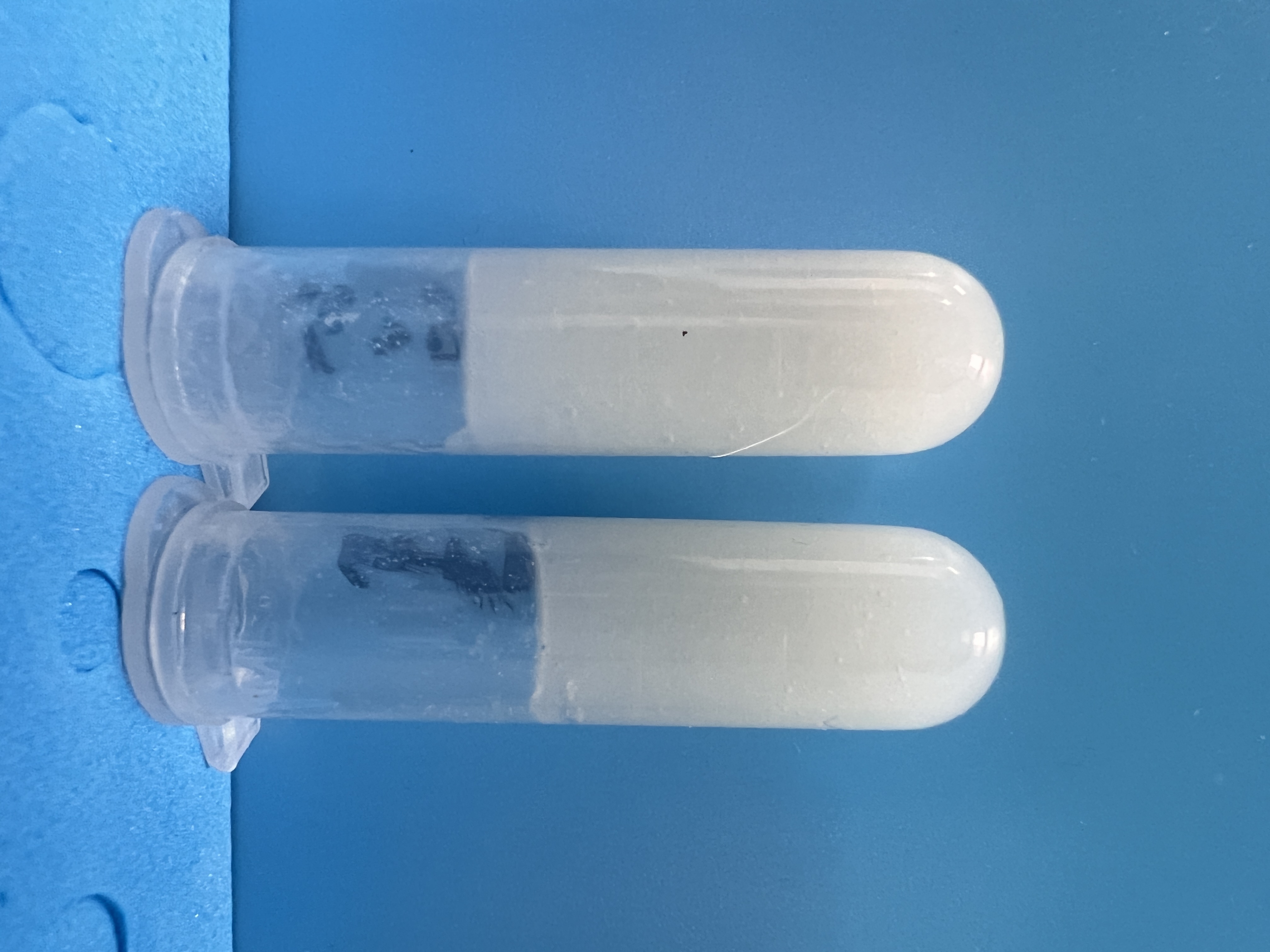


Figure S2. Characterization of the GD hydrogel scaffolds. The formation of a Schiff base bond (DBNC-CH=N-GelMA) between the amino group (GelMA hydrogel) and the aldehyde group (DBNC).


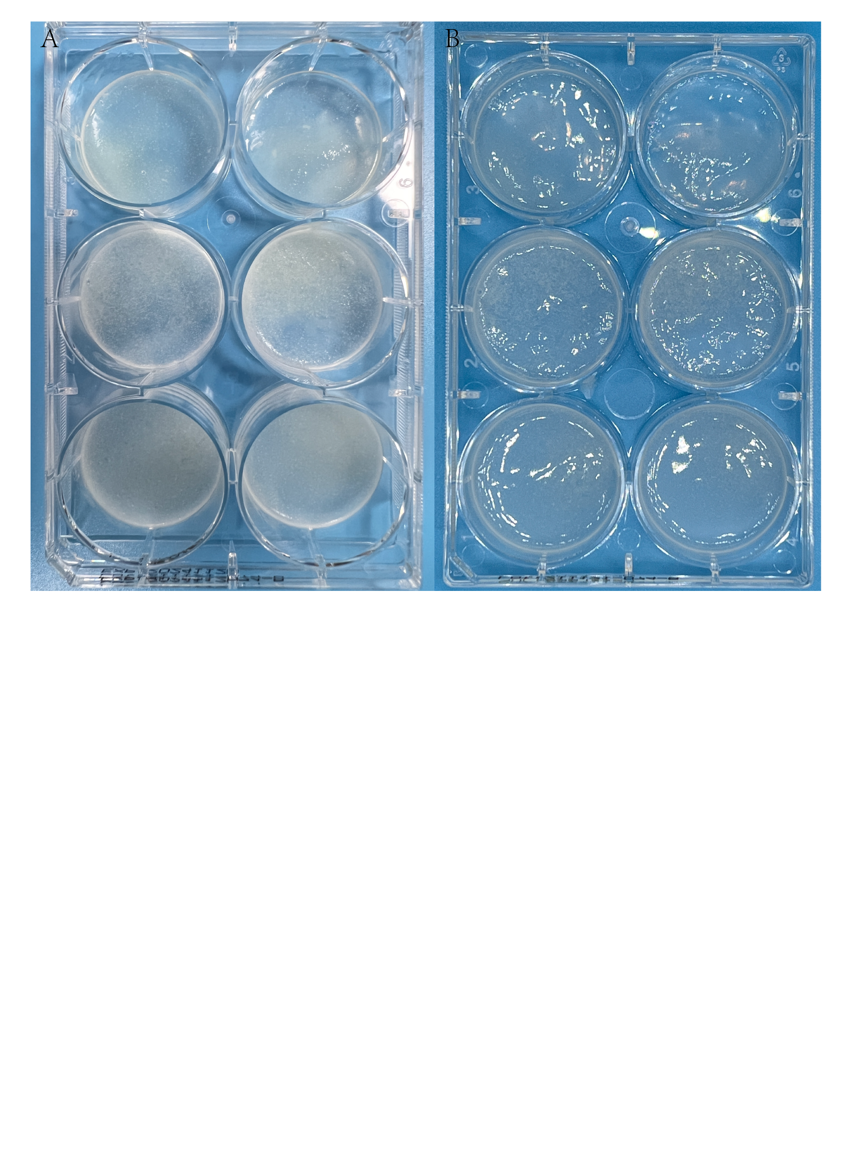


Figure S3. Characterization of the hydrogels with or without Ca^2+^. A: GDAI+ Ca^2+^ hydrogels; B: GDAI hydrogels.


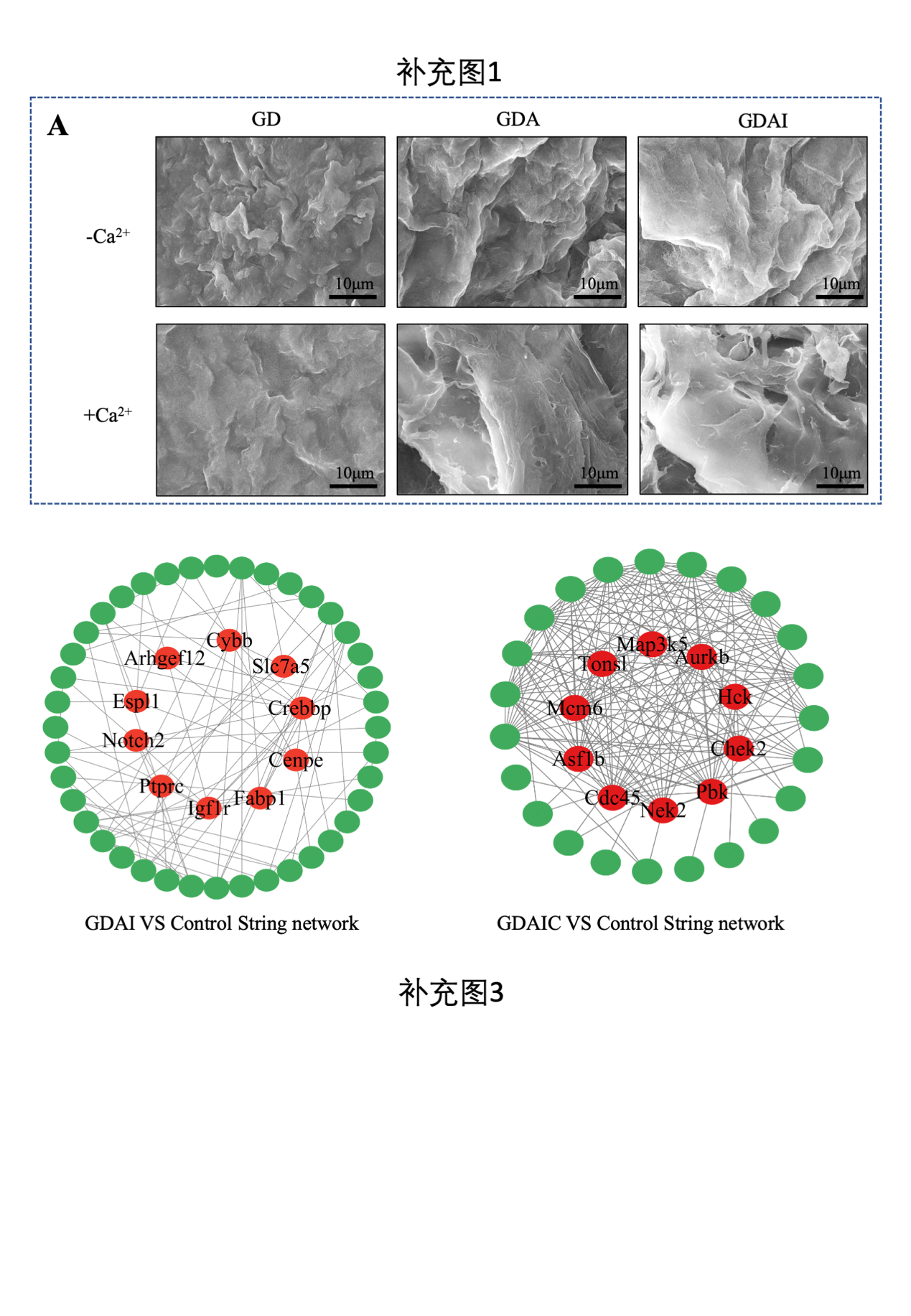


Figure S4. Characterization of the hydrogel scaffolds with or without Ca^2+^.


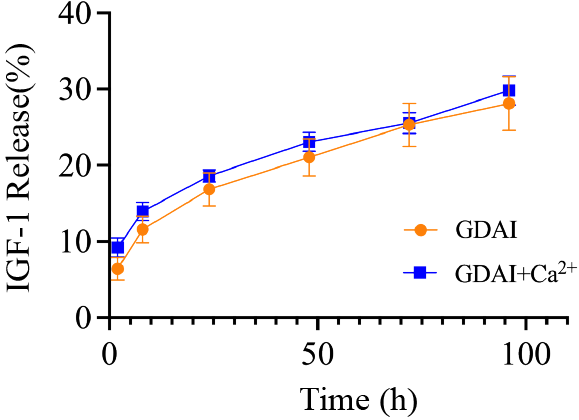


Figure S5. The release rate of IGF-1 in the GDAI and GDAI+Ca^2+^ hydrogels.

Table S1. Primer sequence used for RT-qPCR analysis.

| Genes | Primer sequence（5'-3') | NCBI ID |
| --- | --- | --- |
| GAPDH | | CCTCGTCCCGTAGACAAAATG | | --- | | TGAGGTCAATGAAGGGGTCGT | | NM_008084.2 |
| OSTERIX | | CCTCGTCCCGTAGACAAAATG | | --- | | TGAGGTCAATGAAGGGGTCGT | | [NM_001037632.1](https://www.ncbi.nlm.nih.gov/entrez/viewer.fcgi?db=nucleotide&id=83313664) |
| RUNX2 | | AGGCGTATTTCAGATGATGACACT | | --- | | TAGGTAAAGGTGGCTGGGTAGTG | | [NM_001278483.1](https://www.ncbi.nlm.nih.gov/entrez/viewer.fcgi?db=nucleotide&id=511094001) |
| BMP2 | | AGGCACCCTTTGTATGTGGACT | | --- | | GCCTTAGGGATTTTGGAATTCAC | | NM_007553.3 |
| Sox9 | | GCTGAAGGGCTACGACTGGA | | --- | | TTGCCCATTCTTCACCGACT | | NM_080403.1 |
| ACAN | | AGTGACCCATCTGCTTACCCTG | | --- | | CTGCATCTATGTCGGAGGTAGTG | | [XM_039101034.1](https://www.ncbi.nlm.nih.gov/nuccore/XM_039101034.1) |
| Col 2 | | GAGCGGAGACTACTGGATTGATC | | --- | | GACGTTAGCGGTGTTGGGAG | | [NM_012929.1](https://www.ncbi.nlm.nih.gov/entrez/viewer.fcgi?db=nucleotide&id=6978676) |
| KLF13 | TTGCCAGGGTCTCTACTCTGTCTC  CAGCTGAACTTCTTCTCGCCC | [NM_001109147.1](https://www.ncbi.nlm.nih.gov/entrez/viewer.fcgi?db=nucleotide&id=157820690) |
| RUNX3 | GCTCACAATCACCGTGTTCACC  AGGCTTTGGTCTGGTCCTCTATCT | [NM_001411778.1](https://www.ncbi.nlm.nih.gov/entrez/viewer.fcgi?db=nucleotide&id=-2001204838) |
| Col10a1 | | TTTCTGGGATGCCTCTTGTCA | | --- | | AGATCTTGGGTCATAGTGCTGCT | | [XM_001053056.7](https://www.ncbi.nlm.nih.gov/entrez/viewer.fcgi?db=nucleotide&id=1046877595) |
